# Therapeutic Properties and Underlying Mechanisms of Traditional Chinese Medicine in Managing Rheumatoid Arthritis: An Integrative Review of Clinical and Molecular Findings

**DOI:** 10.1002/ptr.70429

**Published:** 2026-08-18

**Authors:** Jianting Wen, Jian Liu, Lei Wan, Fanfan Wang, Yang Li

**Affiliations:** ^1^ Department of Rheumatology and Immunology First Affiliated Hospital of Anhui University of Chinese Medicine Hefei Anhui China; ^2^ Institute of Rheumatology Anhui Academy of Chinese Medicine Hefei Anhui China; ^3^ Anhui Province Key Laboratory of Modern Chinese Medicine Department of Internal Medicine Application Foundation Research and Development Hefei Anhui China

**Keywords:** clinical study, multi‐target therapy, rheumatoid arthritis, traditional Chinese medicine

## Abstract

Traditional Chinese medicine (TCM) has a long‐standing history in the treatment of rheumatoid arthritis (RA), known as “Bi Zheng,” and offers a comprehensive therapeutic approach characterized by multi‐target and multi‐pathway mechanisms. Modern pharmacological research has demonstrated that bioactive compounds and herbal formulas derived from TCM (e.g., triptolide [TPL] from *Tripterygium wilfordii* and the Xinfeng Capsule [XFC]) effectively interfere with the complex pathogenesis of RA. These interventions modulate diverse pathological processes, including the suppression of pro‐inflammatory cytokines (e.g., TNF‐α, IL‐6, and IL‐1β), induction of apoptosis in hyperplastic synovial cells, and regulation of novel cell death pathways (such as ferroptosis and pyroptosis). Furthermore, TCM components exhibit potent antioxidant properties, inhibit pathological angiogenesis, ameliorate hypercoagulable states, and restore gut microbiota dysbiosis, which is increasingly recognized as a key environmental factor in RA development. Notably, emerging evidence highlights the critical role of non‐coding RNAs (ncRNAs) as mediators of these therapeutic effects. Concurrently, a growing body of clinical evidence from randomized controlled trials (RCTs), cohort studies, and real‐world data mining consistently confirms the significant efficacy and safety of TCM interventions in relieving RA symptoms, improving joint function, and reducing disease activity. By integrating preclinical and clinical findings, a robust evidence chain emerges, validating traditional TCM concepts through modern scientific rigor. Therefore, TCM represents a valuable resource for RA drug discovery and integrative treatment strategies. Further in‐depth investigation is needed into its specific molecular targets, optimal formulations, and long‐term clinical outcomes to facilitate its global translation and application.

## Graphical abstract

The translational research framework integrating TCM for RA. The figure outlines a stepwise framework for the modernization and clinical translation of TCM in RA. Part 1 highlights the inheritance and development of TCM theory, including classical medical texts, traditional herbal knowledge, and syndrome differentiation–based treatment principles. Part 2 summarizes modern basic and scientific research, encompassing in vivo and in vitro experiments, FLS and serum studies, electron microscopy, bulk expression profiling, and molecular detection. Part 3 illustrates data science–driven translational research through database integration, literature mining, data mining, and bioinformatic analysis. Part 4 emphasizes high‐level clinical validation based on RA populations, PBMCs, laboratory indicators, TCM syndrome scores, and outcome‐oriented data analysis. Part 5 presents future‐oriented clinical application strategies, including AI, RNA‐based regulation, nanoparticle drug delivery, precision medicine, and globalization. Overall, the figure depicts the translational progression of TCM for RA from molecular mechanisms to clinical practice.

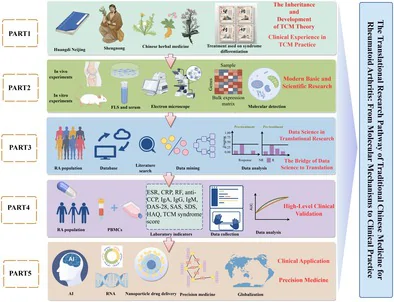

**Abbreviations**
AA: adjuvant arthritis
ACR: American College of Rheumatology
AI: artificial intelligence
AIA: adjuvant‐induced arthritis
AKT: protein kinase B
ALP: alkaline phosphatase
ALT: alanine aminotransferase
AMPK: AMP‐activated protein kinase
APTT: activated partial thromboplastin time
AST: aspartate aminotransferase
bDMARDs: biological DMARDs
bFGF/FGF: basic fibroblast growth factor
BHGZD: Baihu‐Guizhi decoction
BME: bone marrow edema
BUN: blood urea nitrogen
C3/C4: complement component 3/4
CASP/Caspase: cysteine‐aspartic proteases
CCL: chemokine (C‐C motif) ligand
CCP: cyclic citrullinated peptide
CDAI: Clinical Disease Activity Index
ceRNA: competitive endogenous RNA
cGAS‐STING: cyclic GMP‐AMP synthase—Stimulator of interferon genes
CIA: collagen‐induced arthritis
circRNA: circular RNA
CNKI: China National Knowledge Infrastructure
CREA: creatinine
CRP: C‐reactive protein
csDMARDs: conventional synthetic DMARDs
CXCL: chemokine (C‐X‐C motif) ligand
DAMPs: damage‐associated molecular patterns
DAS28: disease activity score in 28 joints
DD: D‐dimer
DMARDs: disease‐modifying antirheumatic drugs
EMS: Er Miao San
ESR: erythrocyte sedimentation rate
FBG/FIB: fibrinogen
FHD: Fangji Huangqi Decoction
FLS: fibroblast‐like synoviocytes
GGT: gamma‐glutamyl transferase
GPX4: glutathione peroxidase
GSDMD: gasdermin D
GSDMD‐NT: GSDMD N‐terminal domain
GSH: Glutathione
HAQ: Health Assessment Questionnaire
HAQ‐DI: HAQ Disability Index
HCQ: hydroxychloroquine
HDMEC: human dermal microvascular endothelial cells
HIF‐1α: hypoxia‐inducible factor 1‐alpha
HPLC: high‐performance liquid chromatography
HQC: Huangqin Qingre Chubi Capsule
hs‐CRP: high‐sensitivity CRP
HUVEC: human umbilical vein endothelial cells
Ig: immunoglobulin
IL: interleukin
JAK: Janus kinase
JBP: Juan Bi Pill
JBQG: Juanbi Qianggu Formula
JNK: c‐Jun N‐terminal kinase
Keap1: Kelch‐like ECH‐associated protein 1
LEF: leflunomide
lncRNA: long non‐coding RNA
LPO: lipid peroxide
m6A: N6‐methyladenosine
MAPK: mitogen‐activated protein kinase
MDA: malondialdehyde
miRNA/miR: microRNA
MMPs: matrix metalloproteinases
MPV: mean platelet volume
MREs: miRNA response elements
MSC‐exo: mesenchymal stem cell exosomes
MTX: methotrexate
MyD88: myeloid differentiation primary response 88
ncRNAs: non‐coding RNAs
NETosis: NETs formation
NETs: neutrophil extracellular traps
NF‐κB: nuclear factor kappa‐light‐chain‐enhancer of activated B cells
NLRP3: NLR family pyrin domain containing 3
Nrf2: nuclear factor erythroid 2‐related factor 2
OPG: osteoprotegerin
PAMPs: pathogen‐associated molecular patterns
PBMCs: peripheral blood mononuclear cells
PCT: plateletcrit
PDW: platelet distribution width
PI3K: phosphoinositide 3‐kinase
PKA: protein kinase A
PLT: platelet count
QRHXD/QRHX: Qingre Huoxue Decoction/Granule
RA: rheumatoid arthritis
RANKL: receptor activator of nuclear factor kappa‐B ligand
RAQOL: rheumatoid arthritis quality of life scale
RCT: randomized controlled trial
RF: rheumatoid factor
RNS: reactive nitrogen species
ROS: reactive oxygen species
SAS: Self‐Rating Anxiety Scale
SASP: senescence‐associated secretory phenotype
SCFAs: short‐chain fatty acids
SDS: self‐Rating Depression Scale
SF‐36: 36‐Item Short Form Health Survey
SIRT1: sirtuin 1
SMYAD: Simiao Yong'an decoction
SOD: superoxide dismutase
STAT: signal transducer and activator of transcription
TAOC: total antioxidant capacity
TCM: traditional Chinese medicine
TGR5: Takeda G protein‐coupled receptor 5
Th cells: T helper cells
TLR: toll‐like receptor
TNF‐α: tumor necrosis factor‐alpha
Tregs: regulatory T cells
tsDMARDs: targeted synthetic DMARDs
TT: thrombin time
TXNIP: thioredoxin‐interacting protein
UA: uric acid
UPLC: ultra‐performance liquid chromatograph
VAS: visual analog scale
VEGF: vascular endothelial growth factor
WBT: Wangbi Tablets
Wnt: wingless‐related integration site
WTD: Wutou decoction
XFC: Xinfeng Capsule

## 1 Introduction

Rheumatoid arthritis (RA) is a systemic autoimmune disorder primarily characterized by symmetric inflammatory polyarthritis, accompanied by morning stiffness and synovial swelling of small joints (Smolen et al. 2016; Cush 2022). Beyond articular manifestations, RA presents a range of extra‐articular involvements, including cardiac complications (e.g., pericarditis and myocarditis), pulmonary disorders (such as interstitial lung disease and pleuritis), and hematological abnormalities (e.g., anemia of chronic disease) (Sullivan and Ascherman 2024; Sun et al. 2022; Park et al. 2022). Chronic inflammation may further lead to progressive joint deformity, functional impairment, and significant disability, severely diminishing patients' self‐perspective and affecting their quality of life (Tański et al. 2022; Zhang, Wang, et al. 2023; Zhang, Zhi, et al. 2023; Zhang, Zhu, and Wu 2023). The global prevalence of RA is estimated at 0.5%–1%, with a two‐ to threefold higher incidence in females than in males (Finckh et al. 2022; Almutairi et al. 2021). Moreover, RA is also acknowledged as a life‐long, incurable disease requiring long‐term medication to maintain disease control. Consequently, its chronicity, complexity, and multisystem nature impose substantial economic burdens on individuals, families, and healthcare systems.

The pathogenesis of RA is a multifaceted process involving intricate interactions among genetic predisposition, environmental triggers, and dysregulated immune responses (McInnes and Schett 2011; D'Orazio et al. 2024). Additionally, it has been shown that RA pathogenesis is strongly influenced by genetic predisposition (Dedmon 2020). Specific risk alleles, notably within the HLA‐DRB1 locus, enable antigen‐presenting cells to present citrullinated self‐antigens to T cells, thereby initiating an aberrant adaptive immune response (Wan et al. 2022). CD4^+^ T cells differentiate into various helper T cell subsets (such as Th1 and Th17 cells), while regulatory T cells (Tregs) often exhibit impaired function and frequency. This imbalance disrupts immune homeostasis and promotes inflammation (Jang et al. 2022). Notably, Th17 cells secrete pro‐inflammatory cytokines [including interleukin (IL)‐17 and IL‐22], which activate synovial fibroblasts and recruit neutrophils, thereby exacerbating joint inflammation (Chen, Cao, et al. 2024; Chen, Chen, et al. 2024). Concurrently, B cells contribute to RA pathogenesis through the production of autoantibodies, such as rheumatoid factor (RF) and cyclic citrullinated peptide (CCP) antibodies (Karmakar and Vermeren 2021; McGrath et al. 2024). These antibodies form immune complexes that deposit in joint tissues, activating the complement cascade and further amplifying local inflammation. Innate immunity also plays an essential role. Specifically, macrophages activated by tumor necrosis factor‐alpha (TNF‐α) and IL‐1 release additional cytokines (such as IL‐6 and IL‐23), establishing a positive feedback loop that enhances Th17 differentiation and sustains inflammatory circuits (Qin et al. 2022). The transformation of fibroblast‐like synoviocytes (FLS) into an aggressive phenotype represents a pivotal step in RA progression. These activated FLS produce matrix metalloproteinases (MMPs) and receptor activator of nuclear factor kappa‐B ligand (RANKL), which directly mediate cartilage degradation and osteoclastogenesis, leading to joint destruction (Cutolo et al. 2022; Zheng et al. 2024). Taken together, these interconnected immune and stromal mechanisms underscore the complexity and chronicity of RA.

Current management of RA relies on a treat‐to‐target strategy employing conventional synthetic disease‐modifying antirheumatic drugs (csDMARDs), biological DMARDs (bDMARDs), and targeted synthetic DMARDs (tsDMARDs) (Smolen et al. 2023). Although these agents have markedly improved clinical outcomes, their utility is often constrained by significant limitations. Primary concerns include systemic immunosuppression, which elevates the risk of serious infections, and organ‐specific toxicities (such as hepatotoxicity, bone marrow suppression, and renal impairment) (Krabbe et al. 2021). Additionally, certain bDMARDs and tsDMARDs have been shown to be associated with increased risks of cardiovascular events and malignancy (Meissner et al. 2023). A substantial proportion of patients exhibit inadequate response due to primary non‐response, loss of efficacy, intolerable side effects, or the high cost of biologic agents, highlighting treatment heterogeneity and the unmet need for personalized therapeutic approaches. These limitations underscore the urgent need to develop novel, safer, and more effective treatment strategies.

Although conventional therapies for RA primarily focus on immunosuppression and symptom management, complementary approaches rooted in traditional Chinese medicine (TCM) are gaining attention for their multi‐target therapeutic potential (Wang, Chang, et al. 2021; Wang, Chen, et al. 2021; Wang, Wu, and Deng 2021; Wang, Liu, et al. 2025; Wang, Ni, et al. 2025; Wang, Pan, et al. 2025; Wang, Wen, et al. 2025; Wang, Zhang, et al. 2025). TCM‐based interventions (including herbal formulas, single herbs, and bioactive compounds) are increasingly studied for their synergistic abilities to mitigate inflammation, modulate immune responses, protect against systemic damage, and improve patients' quality of life (Shi et al. 2020; Wang, Fu, et al. 2024; Wang, Kong, and Li 2024; Wang, Liu, et al. 2024; Wang, Xu, et al. 2024). In TCM theory, RA falls under the category of “Wang Bi (尪痹)” or “Bi Zheng (痹证)”, classifications that reflect both disease progression and clinical manifestations. Modern TCM practice has integrated contemporary medical insights to establish a multi‐stage, syndrome‐based diagnostic framework. During the acute phase, RA typically presents as either dampness‐heat syndrome or cold‐dampness syndrome. The former is treated with heat‐clearing/dampness‐drying herbs, while the latter is managed with warm‐therapy agents to dispel cold and resolve dampness (Chen et al. 2022). As the disease evolves, patterns such as spleen deficiency with dampness accumulation or liver‐kidney deficiency become prominent, necessitating treatments that strengthen the spleen, resolve dampness, and tonify the liver and kidneys. In advanced stages, joint deformities often correlate with phlegm‐stasis binding syndrome, which is addressed through blood‐activating and stasis‐eliminating strategies (Qin et al. 2020), as summarized in Figure 1. Notably, guided by the principle of “Guben Peiyuan” (固本培元, strengthening the foundation and cultivating the source), the Xin'an medical school has made significant contributions to RA treatment. Represented by Professor Liu Jian, this research team identifies “spleen deficiency with dampness excess” (脾虚湿盛) as a central pathological mechanism and advocates “strengthening the spleen and resolving dampness” (健脾化湿) as a core treatment strategy (Liu et al. 2017; Huang et al. 2021; Wen and Liu 2021). Based on this principle, they developed the Xin'an Jianpi Tongbi Formula, which comprises three optimized herbal formulations targeting distinct rheumatic diseases subtypes: the Jianpi Huashi Tongluo Formula [Xinfeng Capsule (XFC), 新风胶囊, Patent ZL201310011369.8] (Wen et al. 2020; Gao et al. 2021), the Jianpi Qingre Tongluo Formula [Huangqin Qingre Chubi Capsule (HQC), 黄芩清热除痹胶囊, Patent ZL201110095718.X] (Liu et al. 2022), and the Jianpi Wenyang Tongluo Formula (Wuwei Wentong Chubi Capsule, 五味温通除痹胶囊, Patent ZL201110095704.8) (Zhang, Liu, et al. 2019; Zhang, Wang, et al. 2019). The development of these modern formulas is deeply grounded in the long‐standing traditional use of a core group of medicinal plants for managing “Bi Zheng.” These formulas have been systematically evaluated from clinical efficacy to molecular mechanisms, demonstrating notable therapeutic benefits in RA. This theoretical framework has guided the selection and application of a specific repertoire of medicinal plants over millennia. The modern scientific investigation of TCM for RA is therefore not an isolated endeavor but a direct inquiry into this rich ethnopharmacological legacy.

**FIGURE 1:**
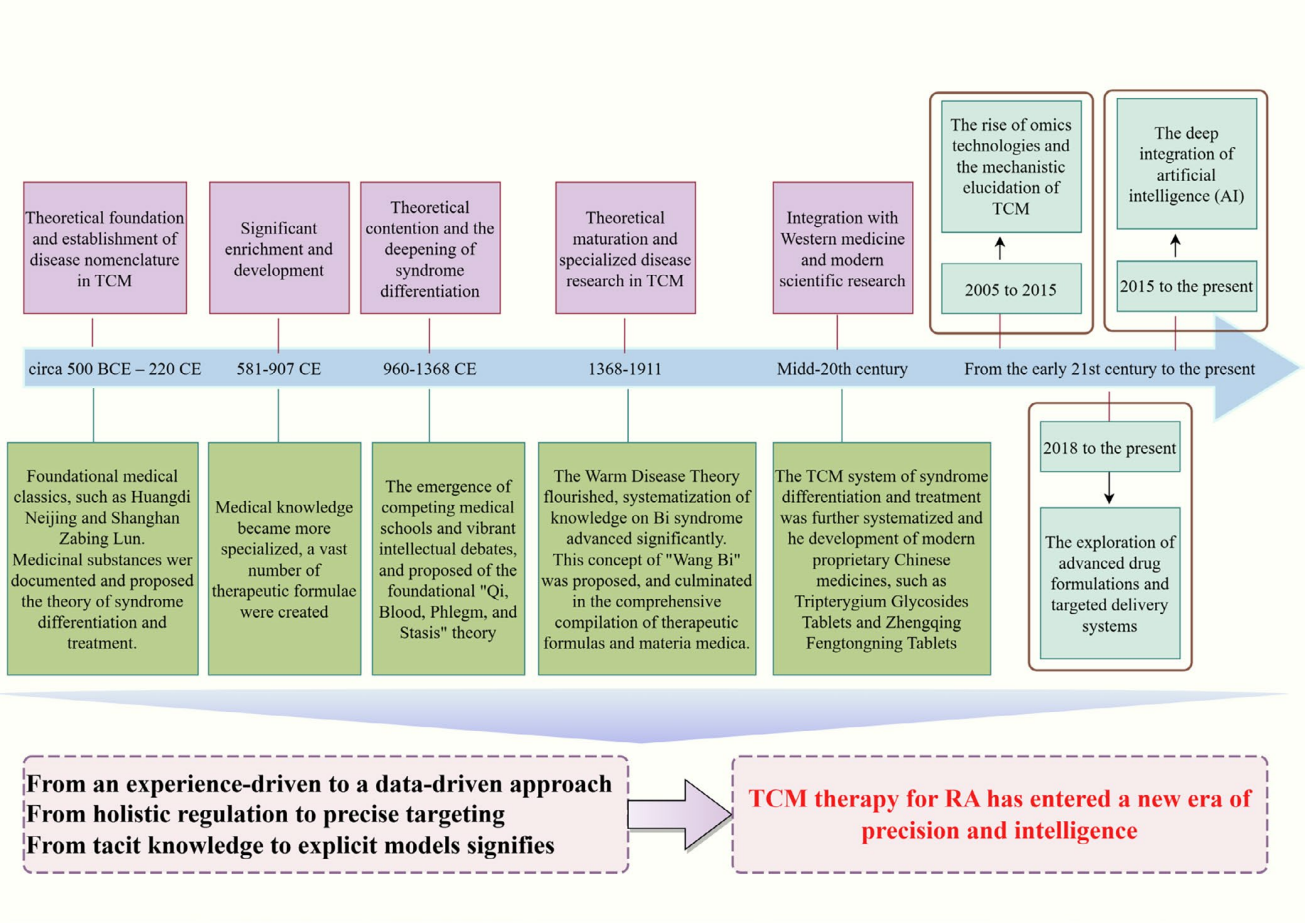
Historical evolution and modern integration of TCM theory and practice in RA. The timeline summarizes the major developmental stages of TCM in the understanding and treatment of RA. It begins with the establishment of disease concepts and syndrome differentiation in early medical classics, followed by the enrichment of therapeutic formulas, the maturation of “Wang Bi” theory, and the progressive systematization of syndrome differentiation and treatment. Since the mid‐20th century, the integration of TCM with Western medicine and modern scientific research has accelerated its modernization. In the 21st century, advances in omics technologies, AI, and targeted delivery systems have further promoted the mechanistic elucidation and precision application of TCM in RA. Overall, the figure illustrates the evolution of TCM for RA from an experience‐driven, holistic framework toward a data‐driven, mechanism‐based, and increasingly precise and intelligent paradigm.

In recent years, TCM, rooted in syndrome differentiation and emphasizing individualized and holistic views, has emerged as a promising therapeutic approach for RA. TCM‐based interventions commonly target multiple pathological processes associated with RA pathogenesis, including suppression of inflammatory responses, restoration of oxidative‐antioxidative imbalance, regulation of various cell death pathways, and modulation of gut microbiota dysbiosis. Mechanistic research on TCM has evolved from a primary focus on anti‐inflammatory and immunomodulatory effects toward a more comprehensive understanding of its role in modulating metabolism and programmed cell death modalities. This multimodal intervention strategy provides a robust scientific foundation for developing novel therapeutic regimens. This review aimed to systematically summarize clinical evidence, elucidate underlying molecular mechanisms, and facilitate subsequent drug development for TCM‐based RA management. Moving forward, it is imperative to clarify the precise mechanisms of TCM interventions using modern technologies, thereby fostering convergence between empirical knowledge and modern medical science. Such efforts will help construct a more complete evidence chain to support the broader application of TCM in RA treatment.

## 2 Ethnopharmacological Relevance and Traditional Use of Medicinal Plants in RA

In TCM theory, RA falls under the category of “Bi Zheng” or “Wang Bi,” which translates to “impediment syndrome” characterized by pain, swelling, and immobility of the joints. RA pathogenesis is traditionally attributed to the invasion of external pathogenic factors—namely Wind (风), Cold (寒), and Dampness (湿)—which obstruct the meridians and the flow of Qi (vital energy) and blood, leading to pain and dysfunction. Over time, this can deplete the body's vital energy, particularly of the liver, kidney, and spleen, resulting in deficiency patterns mixed with excess pathogens.

Over centuries of clinical practice, TCM has offered a core set of medicinal plants for treating “Bi Zheng”. Many of these plants form the basis of the modern formulas and compounds discussed in this review. The therapeutic rationale for these plants in TCM is intrinsically linked to their purported properties. Specifically, for “Cold‐Dampness Bi Syndrome”, herbs with warm and acrid properties are used to warm the meridians and scatter cold, with Wu Tou (*Aconiti Radix*) being a cornerstone herb in classical formulas such as Wutou Decoction (WTD). For Dampness‐Heat Bi Syndrome, herbs with cold and bitter properties are used to clear heat and dry dampness, with Huang Qin (*Scutellariae Radix*) serving as a primary herb in this category and forming the core of HQC. For chronic Bi with Deficiency, herbs that tonify Qi and blood and strengthen the liver and kidney are employed, with Huang Qi (*Astragali Radix*) being widely used for this purpose, as seen in XFC and many other formulas.

Crucially, many of the bioactive monomeric compounds are directly derived from these traditionally used plants. Their molecular mechanisms are elaborated in later sections of this review. Table 1 below provides a representative (non‐exhaustive) list of these plants. The table summarizes their traditional uses and properties in TCM for RA, along with their key bioactive compounds investigated in modern pharmacological studies.

**TABLE 1:** TCM herbs for RA.

TCM herb (latin name)	Traditional TCM property and role in “Bi Zheng”	Key bioactive compounds (discussed in this review)	Modern TCM formula(s) containing the herb
Tripterygium wilfordii (雷公藤, *Leigongteng*)	To dispel wind‐dampness, relieve pain, and reduce swelling. A potent herb for stubborn Bi	TPL, (5R)‐5‐hydroxytriptolide (LLDT‐8)	XFC
Scutellaria baicalensis (黄芩, *Huangqin*)	To clear heat and dry dampness, particularly for Bi syndromes with heat signs (red, swollen, hot joints)	(Baicalin—often a marker compound)	HQC
Paeonia lactiflora (白芍, Baishao)	To nourish blood, soften the liver, and relieve pain. Used to alleviate spasm and pain in joints	PF	WTD
Ligusticum chuanxiong (川芎, Chuanxiong)	To invigorate blood circulation, promote Qi movement, and dispel wind to relieve pain	TMP	(Cited as the source of the monomer TMP)
Sinomenium acutum (青风藤, Qingfengteng)	To dispel wind‐dampness and unblock the collaterals to relieve pain	Sinomenine	(Cited as the source of the monomer Sinomenine and its derivative SINX)
Epimedium brevicornum (淫羊藿, Yinyanghuo)	To tonify kidney Yang and strengthen tendons and bones, addressing the deficiency aspect of chronic Bi	ICA	(Cited as the source of the monomer ICA)
Rheum palmatum (大黄, Dahuang)	To drain heat, purge fire, and invigorate blood. Used in Bi syndromes with excessive heat and constipation	EMO	(Cited as the source of the monomer EMO)
Astragalus membranaceus (黄芪, Huangqi)	To tonify Qi and strengthen the exterior, used for Bi with Qi deficiency and to promote tissue healing	(Astragalosides—often a marker compound)	XFC; WTD
Coix lacryma‐jobi (薏苡仁, Yiyiren)	To drain dampness and strengthen the spleen, addressing the dampness pathogen in Bi	CSO	XFC; HQC
Clematis chinensis (威灵仙, Weilingxian)	To dispel wind‐dampness and unblock the collaterals, particularly for pain and numbness	(Component of compound formulas)	HQC
Aconitum carmichaelii (附子, Fuzi)	To dispel cold and relieve pain, warm the meridians, and tonify fire (Yang)	(Studied as the herb Fuzi for gut microbiota)	(Cited as the monomer herb Fuzi in gut microbiota studies)
Prunus persica (桃仁, Taoren)	To invigorate blood circulation, remove blood stasis	/	HQC
Gardenia jasminoides (栀子, Zhizi)	To clear heat and drain fire, cool blood and detoxify	/	HQC
Scolopendra subspinipes (蜈蚣, Wugong)	To extinguish wind and relieve convulsion, unblock collaterals and relieve pain	/	XFC
Aconitum kusnezoffii/A. carmichaelii (乌头, Wutou)	To dispel wind‐dampness, warm the meridians, and relieve pain	/	WTD
Ephedra sinica (麻黄, Mahuang)	To induce sweating and dispel wind‐cold, promote urination to reduce edema, unblock collaterals	/	WTD
Glycyrrhiza uralensis (甘草, *Gancao*)	To tonify the spleen and Qi, clear heat and detoxify, relieve spasm and pain, harmonize other herbs	/	WTD

This ethnopharmacological foundation provides the essential link. It connects centuries of observational clinical experience with contemporary molecular inquiry. The plants listed above are not random selections but are the very sources of the compounds, such as triptolide (TPL), paeoniflorin (PF), tetramethylpyrazine (TMP), icariin (ICA), and emodin (EMO). The mechanisms of these compounds in modulating inflammation, apoptosis, oxidative stress, and other RA‐related pathways are detailed in the Section 5. For instance, the potent anti‐inflammatory and immunomodulatory effects of TPL, a constituent of *Tripterygium wilfordii*, are now scientifically rationalized the traditional use of this herb for severe, swollen joints. Similarly, the traditional application of Scutellaria baicalensis to “clear heat” aligns with the modern understanding of its compounds inhibiting pro‐inflammatory cytokines.

Therefore, the subsequent discussions not only describe natural product pharmacology, but also directly translate and scientifically validate a well‐established traditional ethnopharmacopeia at the molecular level. This integrative approach bridges traditional wisdom with modern scientific rigor, which is a core principle of ethnopharmacology. Importantly, the relationship between traditional indications and modern pharmacological findings is not merely symbolic but represents a potentially interpretable correspondence between different knowledge systems. In RA, the TCM concept of “heat” is often associated with redness, swelling, and inflammatory overactivation, which may correspond to excessive cytokine production, immune cell infiltration, and abnormal angiogenesis in modern biomedical terms. Likewise, “dampness” in TCM may be linked to edema, turbidity, and metabolic imbalance in modern terms; “stasis” in TCM may reflect impaired microcirculation, persistent synovial hyperplasia, and tissue remodeling in modern terms. From this perspective, the traditional use of herbs and formulas for clearing heat, resolving dampness, and removing stasis may help explain the anti‐inflammatory, anti‐angiogenic, anti‐fibrotic, and microenvironment‐modulating effects of many TCM interventions in RA.

## 3 Literature Search Strategy

A comprehensive literature search was conducted to identify relevant studies on TCM for the treatment of RA. In PubMed, Medical Subject Headings (MeSH terms; e.g., “Arthritis, Rheumatoid/drug therapy,” “Medicine, Chinese Traditional,” “Herbal Medicine,” and “Drugs, Chinese Herbal”) combined with free‐text terms were utilized to maximize retrieval. Similarly, for Web of Science, the MeSH‐aligned search was adapted using equivalent terminology within the Core Collection databases, focusing on title, abstract, and keyword fields. For the China National Knowledge Infrastructure (CNKI) database, CJFD Subject Terms [e.g., “类风湿关节炎” (RA), “Bi Zheng,” “Wang Bi,” “中医药” (TCM), and “中药治疗” (Chinese herbal medicine therapy)], alongside free‐text queries were employed to capture region‐specific publications.

Furthermore, studies with suboptimal methodological rigor were excluded from the analysis. These included those lacking appropriate controls, quality control for herbal extracts or products, sufficient description of experimental procedures, clear methods for establishing animal models, complete specification of formulation components, and adequate mechanistic investigation. Relevant data were obtained through a systematic literature search. This search may provide a foundation for integrating clinical and experimental research on RA treatment using TCM compounds, individual medicinal plants, and active constituents.

## 4 Pathogenesis of RA

RA pathogenesis is intricately multifaceted, with a dysregulated and persistent inflammatory response standing as its most fundamental and core mechanism (Liu, Wang, Huang, and Sun 2023; Liu, Wang, Li, et al. 2023). This pervasive inflammatory response is not merely a symptomatic consequence but the primary driver that initiates and perpetuates the entire disease process (Mihaylova et al. 2024). It has been shown that the cascade begins with a breach of immune tolerance, leading to activation of innate immune cells (e.g., macrophages, dendritic cells) and subsequent recruitment of adaptive immune cells (particularly T and B lymphocytes) (Komatsu and Takayanagi 2022). This cellular interplay creates a self‐sustaining inflammatory milieu within the synovium. Critically, activated macrophages and fibroblasts overproduce key pro‐inflammatory cytokines, most notably TNF‐α, IL‐6, and IL‐1. These cytokines act as potent molecular messengers, promoting synovial inflammation and hyperplasia, as well as the production of destructive enzymes such as MMPs and cathepsins (Yang et al. 2023; Chang and Tang 2024). Consequently, this leads to hallmark articular destruction, erosion of cartilage and bone (Kemble and Croft 2021). Furthermore, the inflammation becomes systemic, thus contributing to comorbidities. Accumulating evidence has revealed that long non‐coding RNA (lncRNAs) play critical regulatory roles in the inflammatory pathogenesis of RA (Golestanifar et al. 2025). For instance, lncRNA SNHG14 functions as a ceRNA that sponges microRNA (miR)‐17‐5p, which upregulates MINK1, activates the c‐Jun N‐terminal kinase (JNK) signaling pathway, and subsequently enhances inflammatory responses in macrophages, thereby contributing to RA progression (Zhang, Lei, and Li 2021; Zhang, Ma, et al. 2021). Liu, Li, et al. (2025), Liu, Wang, et al. (2025), and Liu, Zhang, et al. (2025) have also revealed that overexpression of lncRNA MEG3 could alleviate inflammatory phenotypes of RA synovial fibroblasts by competitively sponging miR‐93‐5p to promote SMAD7 expression. Xie et al. (2023) have verified that LINC00324 is upregulated in RA and may exaggerate inflammation by targeting miR‐10a‐5p through the nuclear factor kappa‐light‐chain‐enhancer of activated B cells (NF‐κB) signaling pathway. Similarly, circular RNAs (circRNAs) are also emerging as key regulators in the inflammatory pathogenesis of RA. Consistently, Niu, Chen, et al. (2025) and Niu, Li, et al. (2025) have recently demonstrated that circ_0001715 sequesters miR‐326 to regulate inflammation in RA‐FLS cells through the toll‐like receptor (TLR)4/NF‐κB axis.

Hence, targeting this core inflammatory mechanism remains the cornerstone of RA therapy, reflecting its central role in disease etiology and progression.

Apoptosis, or programmed cell death, is critically dysregulated in the pathogenesis of RA, contributing significantly to both synovial inflammation and joint destruction (Liu and Pope 2003). A paradoxical imbalance characterizes apoptotic in RA: immune effector cells (such as activated T lymphocytes and macrophages) exhibit marked resistance to apoptosis, whereas structural cells including chondrocytes and osteoblasts undergo excessive apoptosis (Firestein et al. 1995; Yang et al. 2020). This resistance leads to the accumulation of long‐lived immunocompetent cells in the synovium, perpetuating inflammation and driving synovial hyperplasia. In contrast, excessive apoptosis of structural cells is mediated through death receptors (e.g., Fas and TNF receptor superfamily activation), mitochondrial dysfunction, and oxidative stress, leading to cartilage degradation and bone erosion. Furthermore, defective clearance of apoptotic bodies impairs efferocytosis, leading to secondary necrosis and the release of neo‐autoantigens, which can amplify autoimmune responses. Thus, the dysregulation of apoptotic pathways represents a key mechanism in RA pathogenesis and highlights potential therapeutic strategies aimed at restoring apoptotic homeostasis (Zhang, Liu, et al. 2019; Zhang, Wang, et al. 2019). As has been evidenced previously, the regulation of apoptosis involves multiple signaling pathways and molecular mechanisms in RA pathology. ncRNAs in RA have garnered increasing attention in this context (Wen et al. 2025). For example, lncRNA HOTTIP regulates TLR4 expression by recruiting MLL1, leading to TLR4 promoter methylation, thereby inducing cell apoptosis in RA (Wang, Fu, et al. 2024; Wang, Kong, and Li 2024; Wang, Liu, et al. 2024; Wang, Xu, et al. 2024). In another study, N6‐methyladenosine (m6A)‐mediated lncRNA MAPKAPK5‐AS1 has been shown to induce apoptosis by regulating the miR‐146a‐3p/sirtuin 1 (SIRT1)/NF‐κB axis in RA (Wen et al. 2023). Additionally, signaling pathways play a crucial role in regulating cell apoptosis in RA. The NF‐κB signaling pathway, for example, significantly influences RA‐FLS apoptosis; inhibiting this pathway can promote FLS apoptosis and subsequently alleviate RA symptoms (Zhang, Chen, et al. 2024; Zhang, Weng, et al. 2024).

Oxidative stress, characterized by an imbalanced redox state due to excessive reactive oxygen species (ROS) production and/or compromised antioxidant defenses, plays a pivotal role in the pathogenesis of RA (López‐Armada et al. 2022). This persistent oxidative burden is not merely a secondary consequence of chronic inflammation but is increasingly recognized as a critical driver of disease initiation and progression. A primary source of ROS within the inflamed synovium is the activated phagocytic cells (notably neutrophils and macrophages), which generate a superoxide burst via the NOX2 complex (Zamudio‐Cuevas et al. 2022). Furthermore, dysfunctional mitochondrial electron transport chains in FLS and T cells contribute remarkably to ROS generation (Kaur et al. 2021; Lee et al. 2021). Crucially, it has been shown that ROS act as potent secondary messengers, modulating key pro‐inflammatory signaling pathways. They activate transcription factors [such as NF‐κB and activator protein‐1 (AP‐1)], thereby amplifying the expression of cytokines (TNF‐α, IL‐1β, and IL‐6), chemokines, and MMPs. This perpetuates synovitis, drives hyperplastic synovial fibroblast survival, and facilitates cartilage and bone erosion (Wang, Wang, et al. 2022; Wang, Zhou, et al. 2022). LncRNAs have been shown to regulate oxidative stress responses, thereby playing a significant role in RA progression. For example, Cai et al. (2024) have reported that low‐expressed lncRNA DANCR may regulate the Keap1‐nuclear factor erythroid 2‐related factor 2 (Nrf2)/ARE pathway and suppress oxidative responses in RA patients. Similarly, Sun et al. (2022) have indicated that overexpression of LINC00638 can inhibit oxidative stress in RA‐FLS by activating the Nrf2/HO‐1 pathway.

Pyroptosis is a form of programmed, pro‐inflammatory cell death distinct from apoptosis, which has emerged as a critically pathogenic mechanism in the initiation and perpetuation of RA (Chadha et al. 2020). It plays a significant role in RA as it can directly fuel the autoinflammatory milieu and drive tissue destruction characteristic of RA (Wu et al. 2023). The underlying mechanism is primarily mediated by the cleavage of Gasdermin D (GSDMD) by inflammatory caspases, forming plasma membrane pores. These pores lead to cell swelling, lytic death, and the explosive release of pro‐inflammatory cytokines (e.g., IL‐1β and IL‐18). In RA, pyroptosis is frequently initiated within the synovium via the canonical NLR family pyrin domain‐containing 3 (NLRP3) inflammasome pathway, activated by diverse damage‐associated molecular patterns (DAMPs) and pathogen‐associated molecular patterns (PAMPs). However, pyroptotic signaling in RA is not limited to NLRP3 alone. Emerging evidence suggests that other inflammasomes (such as AIM2 and NLRC4) may also participate in synovial inflammation and immune dysregulation, thereby broadening the mechanistic framework of inflammasome‐driven pathology in RA (Yang and Liu 2022; Jiang, Ji, et al. 2023; Jiang, Wang, et al. 2023). The consequential release of IL‐1β and IL‐18 potently activates synovial fibroblasts, endothelial cells, and infiltrating immune cells, creating a vicious, self‐amplifying cycle of inflammation (Demarco et al. 2022). Furthermore, the pyroptotic death of synovial macrophages and fibroblasts not only releases more DAMPs, which perpetuate inflammasome activation. It also directly contributes to the synovitis and tissue damage in RA (Zhai et al. 2022; Zhang, Wang, et al. 2023; Zhang, Zhi, et al. 2023; Zhang, Zhu, and Wu 2023). Accumulating studies have demonstrated that ncRNAs are closely linked to the regulation of pyroptosis in RA. For example, studies have revealed a close association between ncRNAs and pyroptosis in RA. Specifically, circ_0000175 has been shown to induce pyroptosis and trigger inflammatory responses during RA progression through the miR‐31‐5p/GSDME axis (Xin et al. 2024). Another investigation has identified circ_0044235 as a regulator of NLRP3‐mediated pyroptosis via the miR‐135b‐5p‐SIRT1 axis (Chen et al. 2021). Additionally, recently recognized forms of regulated cell death (particularly PANoptosis and cuproptosis) should also be considered in the evolving context of RA pathogenesis (Lu et al. 2026; Chang et al. 2023). PANoptosis integrates key features of pyroptosis, apoptosis, and necroptosis into a coordinated inflammatory cell death program. By contrast, cuproptosis represents a copper‐dependent form of cell death linked to mitochondrial metabolism. Although less clearly defined than pyroptosis and ferroptosis, these emerging mechanisms may further expand our understanding of how inflammatory and metabolic stress contribute to synovial injury and joint destruction. These findings collectively highlight these molecular axes as novel therapeutic targets for RA.

Ferroptosis is an iron‐dependent form of regulated cell death characterized by lethal lipid peroxidation, which is increasingly recognized as a critical contributor to the pathogenesis of RA (Zhao, Tang, et al. 2023; Zhao, Yu, et al. 2023). Glutathione (GSH) depletion and glutathione peroxidase (GPX4) inactivation are central to its mechanism, leading to ROS accumulation and oxidative membrane damage (Zhao, Chen, et al. 2022; Zhao, Wei, et al. 2022; Zhao, Yang, et al. 2022). In the RA synovial microenvironment, aberrant iron metabolism and elevated intracellular iron levels further exacerbate lipid peroxidation (Sun, Liu, He, et al. 2025; Sun, Liu, Wang, et al. 2025). Moreover, synovial fibroblasts and macrophages, key effector cells in RA, are particularly susceptible to ferroptosis, which disrupts synovial tissue homeostasis and amplifies inflammatory responses (Piao et al. 2024). Additionally, ferroptosis may synergize with other inflammatory pathways to enhance tissue degradation and oxidative stress, including those mediated by NF‐κB and AMP‐activated protein kinase (AMPK). For instance, it has been shown that MD2 deficiency ameliorates RA in mice via suppressing ferroptosis induced by the MAPK and NF‐κB signaling pathways. Recently, the LKB1‐AMPK signaling has been shown to be essential for protecting RA‐FLS against ferroptosis (Zhou, Liu, et al. 2025; Zhou, Wu, et al. 2025; Zhou, Yang, et al. 2025). Targeting ferroptosis represents a potential therapeutic strategy for RA. This can be achieved through iron chelation, antioxidant administration, or GPX4 activation, which can ameliorate synovial inflammation and halt RA progression.

Angiogenesis, defined as the formation of new blood vessels from pre‐existing vasculature, is a fundamental and early event in RA pathogenesis that contributes significantly to disease progression and chronicity (Wang, Chang, et al. 2021; Wang, Chen, et al. 2021). The hyperplastic synovial pannus tissue is markedly hypoxic and is a hallmark of RA (Lee et al. 2024). This hypoxic environment triggers a robust angiogenic response, primarily driven by the upregulation of potent pro‐angiogenic factors [such as vascular endothelial growth factor (VEGF), angiopoietins, and basic fibroblast growth factor (bFGF)]. This neovascularization is an active process that fuels the vicious cycle of RA, not merely a passive consequence of inflammation (Li et al. 2023). Beyond sustaining inflammation, angiogenesis supports the invasive growth of the pannus itself. By providing oxygen and nutrients, the expanding vascular network enables the pannus to behave in a tumor‐like manner and progressively erode joint structures (Zhao et al. 2025). Consequently, angiogenesis represents a pivotal interface between synovial hyperplasia, chronic inflammation, and tissue destruction (Huo et al. 2025). More recently, ncRNAs have been identified as new regulators of vascular function and angiogenesis in RA. Liu, Wang, Huang, and Sun (2023) and Liu, Wang, Li, et al. (2023) have revealed that lncRNA HOTAIR can activate the phosphoinositide 3‐kinase (PI3K)/AKT pathway through the miR‐126‐3p/PIK3R2 regulatory axis, thereby contributing to synovial angiogenesis in RA. Meanwhile, Yu et al. (2023) have indicated that lncRNA Xist binds to GATA1 and upregulates CCN6, which promotes RA pathogenesis by modulating the angiogenic activity of RA‐FLS.

A pronounced hypercoagulable state is increasingly recognized as a critical intermediary in RA pathogenesis. It creates a vicious cycle of inflammation and thrombosis that exacerbates RA progression (Fazal et al. 2024). This imbalance in pro‐coagulant and anticoagulant mechanisms promotes microvascular thrombosis and fibrin deposition within the synovium, contributing to local hypoxia. Hypoxia, in turn, further amplifies the hypercoagulable state through hypoxia‐inducible factor 1‐alpha (HIF‐1α) activation, fueling the invasive growth of the synovial pannus (Qiang et al. 2022). Additionally, platelets are activated by immune complexes and cytokines. They become active participants in inflammation by releasing chemokines and forming pro‐inflammatory aggregates with leukocytes in RA (Zhang et al. 2016). In a bibliometric and retrospective study, Wang, Liu, Fang, Li, et al. (2023) and Wang, Liu, Fang, et al. (2023) have identified hypercoagulability as a research hotspot in RA; they highlight strong correlations between inflammatory markers [erythrocyte sedimentation rate (ESR) and C‐reactive protein (CRP)] and coagulation parameters (FBG) with disease activity assessed via Chinese Patient‐Reported Index for Rheumatoid Arthritis (CPRI‐RA), suggesting their roles as independent risk factors.

Neutrophil extracellular traps (NETs) are web‐like structures composed of decondensed DNA and antimicrobial proteins released by activated neutrophils, playing a crucial role in RA pathogenesis (Frade‐Sosa and Sanmartí 2023). Their formation, a process called NETosis, is enhanced in the RA synovial microenvironment by autoantibodies, cytokines (e.g., IL‐8, TNF‐α), and immune complexes. NETs have been shown to contribute to RA pathogenesis through several mechanisms. Specifically, they can directly activate FLS, promote inflammatory cytokine release, and damage endothelial cells, thereby exacerbating synovitis and angiogenesis (Zhao et al. 2021; Chen, Cao, et al. 2024; Chen, Chen, et al. 2024). Moreover, NETs also exhibit impaired clearance, leading to prolonged inflammation and tissue injury (Carmona‐Rivera et al. 2025). A previous study has found that NETs promote Tfh cell differentiation in RA by inducing IL‐6 production from FLS via the TLR9/myeloid differentiation primary response 88 (MyD88)/NF‐κB pathway, thereby accelerating RA progression. Another study has revealed that NETs promote osteoclastogenesis and bone erosion in RA by altering the RANKL/osteoprotegerin (OPG) balance through the TLR4 and TLR9 signaling (Wang, Fu, et al. 2024; Wang, Kong, and Li 2024; Wang, Liu, et al. 2024; Wang, Xu, et al. 2024). Notably, NETosis represents a critical interface between innate immunity and autoimmune dysregulation. Therefore, it is a promising therapeutic target for interrupting key pathological pathways in RA.

Cellular senescence, a state of irreversible cell cycle arrest driven by stress and damage signals, is increasingly recognized as a key pathogenic mechanism in RA (Wen et al. 2025). In the RA synovium, various cell types (including FLS and immune cells) undergo accelerated senescence in response to chronic inflammation, genomic stress, and oxidative damage (Xu, Li, Liu, et al. 2024; Xu, Li, Wang, et al. 2024). These senescent cells are not inert. Instead, they adopt a pro‐inflammatory phenotype known as the senescence‐associated secretory phenotype (SASP). The SASP involves the robust secretion of cytokines, chemokines, MMPs, and growth factors (e.g., IL‐6, IL‐8, CCL2, and MMP‐3), which drive cartilage degradation and promote RA progression (Fang, Liu, and Wan 2023; Fang, Liu, Xin, et al. 2023). Notably, senescent RA‐FLS exhibit hyperproliferative and invasive properties, directly contributing to pannus formation and joint destruction. As has been evidenced previously, four key genes (MMP1, CCL7, CXCL1, and HK3) are found upregulated in senescent macrophages, contributing to a pro‐inflammatory environment conducive to RA progression (Chen, Guo, Wu, et al. 2025; Chen, Xu, Chen, et al. 2025; Chen, Xu, Zhang, and Xia 2025). Moreover, single‐cell RNA sequencing analysis has revealed that CCN3 is predominantly expressed by inflammatory fibroblast subpopulations (Tokuhiro et al. 2024). As indicated by subsequent experiments, CCN3 is highly expressed in RA and promotes cellular senescence and osteoclast formation. These features make it a key factor in RA pathogenesis.

Emerging evidence underscores a critical role for gut microbiota dysbiosis in RA initiation and progression (Lin et al. 2023). The gut‐joint axis proposes that alterations in the composition and function of intestinal microbial communities can systemically influence autoimmunity and joint inflammation (Zhao, Chen, et al. 2022; Zhao, Wei, et al. 2022; Zhao, Yang, et al. 2022). Dysbiosis in RA patients is characterized by reduced microbial diversity and expansion of pro‐inflammatory species (e.g., *Prevotella copri* ). This dysbiosis disrupts intestinal barrier integrity, promoting microbial translocation and activation of mucosal immunity. These processes facilitate the presentation of arthritogenic antigens. They also promote molecular mimicry, where microbial peptides cross‐react with self‐antigens and trigger autoantibody production (Zaiss et al. 2021). The ensuing systemic immune activation—characterized by increased levels of pro‐inflammatory cytokines and autoantibodies—ultimately targets the synovium and amplifies local inflammation and joint destruction in RA (Chasov et al. 2024). Emerging evidence indicates that gut‐derived exosomes and bacterial extracellular vehicles (EVs) serve as key mediators within the gut–joint axis. These vehicles act as carriers of microbiota‐derived signals that extend beyond the intestinal environment (Niu, Chen, et al. 2025; Niu, Li, et al. 2025). They encapsulate diverse bioactive cargos (including proteins, lipids, metabolites, and ncRNAs), enabling them to modulate systemic immune responses and facilitate inter‐organ communication (Chronopoulos and Kalluri 2020). Under dysbiotic conditions, EVs may promote intestinal barrier disruption, enhance systemic inflammatory signaling, and contribute to synovial immune activation in RA (Elmi et al. 2016). Therefore, gut‐derived exosomes represent a novel mechanistic link connecting gut microbiota dysbiosis with extraintestinal immune dysregulation and joint inflammation.

The pathogenesis of RA is driven by a dysregulated immune response; key mechanisms include overexpression of pro‐inflammatory cytokines, dysregulation of programmed cell death pathways (apoptosis, pyroptosis, and ferroptosis), oxidative stress, aberrant angiogenesis, and a hypercoagulable state. Additionally, NETs, cellular senescence with a pro‐inflammatory SASP, and gut microbiota dysbiosis further contribute to systemic autoimmunity and joint destruction. These interconnected processes drive a cycle of inflammation, reflecting the multifaceted nature of RA pathogenesis (Figure 2).

**FIGURE 2:**
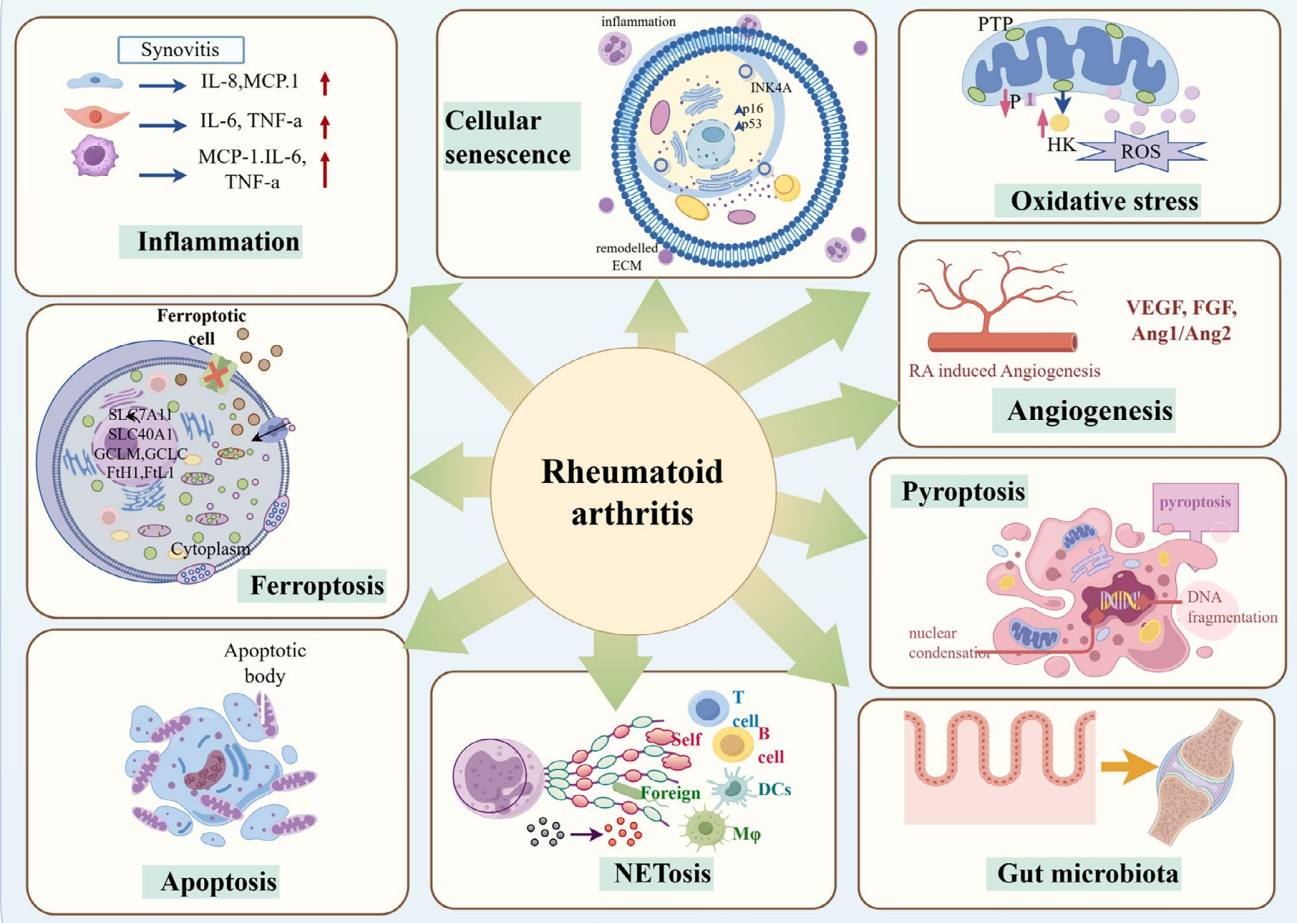
Schematic representation of the key pathological mechanisms involved in RA. RA is driven by a complex and interconnected pathogenic network involving multiple biological processes. These processes include inflammation (characterized by synovitis and increased production of pro‐inflammatory cytokines and chemokines), oxidative stress (associated with mitochondrial dysfunction and excessive ROS generation), angiogenesis (mediated by pro‐angiogenic factors such as VEGF, FGF, and angiopoietins), and regulated cell death pathways (including apoptosis, pyroptosis, and ferroptosis). Additionally, NETosis, cellular senescence, and gut microbiota dysbiosis further contribute to immune dysregulation, synovial injury, and joint destruction. Taken together, these mechanisms form a multidimensional pathological framework underlying RA progression.

## 5 Experimental Study on the Treatment of RA With TCM

Accumulating evidence has demonstrated that TCM plays a crucial role in the management of RA, particularly in reducing the adverse effects of conventional Western drugs and improving patients' quality of life. TCM exerts its therapeutic effects through multifaceted mechanisms, including suppression of inflammatory responses, modulation of various forms of cell death, regulation of gut microbiota homeostasis, and inhibition of angiogenesis (Table 2, Figure 3, and Figure 4). Notably, recent research has also highlighted the involvement of ncRNAs and protein modifications in these processes (Figure 5). Substantial progress in cellular and animal models has provided compelling mechanistic insights into the efficacy of TCM in RA treatment.

**TABLE 2:** Experimental study on the therapeutic effects of TCM for RA.

Mechanism	TCM	Herbal composition	Models	Related targets	Effect
Inflammation	TPL	NA	RA‐FLS and CIA rat model	LNCRNA RP11‐83J16.1/URI1/β‐catenin	TPL decreases RA‐FLS inflammation and presents a therapeutic effect in CIA model via inactivating lncRNA RP11‐83J16.1 mediated URI1 and β‐catenin signaling
	(5R)‐5‐hydroxytriptolide (LLDT‐8)	NA	RA‐FLS	LncRNA WAKMAR2/miR‐4478/E2F1/p53	The inflammation inhibitory effects of LLDT‐8 on RA FLS were dependent on WAKMAR2/miR‐4478/E2F1/p53 axis
	JBQG	Psoralea corylifolia, Drynaria fortunei, Epimedium brevicornu, Sinomenium acutum, and Angelica sinensis	Synovial tissues of RA patients, RA‐FLS and CIA rat model	LncRNA ITSN1‐2/miR‐2683‐3p/PELI3/RIP2	JBQG exerts potent anti‐arthritic effects in RA therapy through dual regulatory mechanisms targeting the lncRNA ITSN1‐2/miR‐2683‐3p/PELI3/RIP2 axis
	TMP	NA	RA‐FLS	Hsa_circ_0005178/HIF‐1α/IL‐6	TMP might block RA‐FLS injury partly via regulating the HIF‐1α‐circCDC42BPB pathway
	TPL	NA	RA patients and RA‐FLS	hsa‐circ‐0003353/miR‐31–5p/CDK1	TPL repressed the inflammatory response of RA‐FLSs by mediating the expression of the circ0003353/miR‐31–5p/CDK1 axis
	Dioscin	NA	RA‐FLS	Circ_0008267/miR‐942‐5p/FKBP5	Dioscin suppressed the inflammatory response in RA‐FLS through circ_0008267/miR‐942‐5p/FKBP5 axis
	Clematichinenoside AR (CAR)	NA	RA‐FLS and CIA mice	CircPTN/miR‐145‐5p/FZD4	CAR blocked the circPTN/miR‐145‐5p/FZD4 signal axis by combining with FZD4 and improved RA pathology
	PF	NA	RA‐FLS	Hsa_circ_009012/miR‐1286/TLR4/NLRP3	PF inhibits the inflammation of RA‐FLS via mediating the hsa_circ_009012/miR‐1286/TLR4/NLRP3 axis
	PF	NA	RA synovial tissues and RA‐FLS	Circ‐FAM120A/miR‐671–5p/MDM4	PF inhibited RA‐FLS inflammation by mediating the circ‐FAM120A/miR‐671–5p/MDM4 pathway
	HQC	Scutellariae Radix, Coicis Semen, Persicae Semen, Gardeniae Fructus, and Clematidis Radix et Rhizoma	RA‐PBMCs co‐cultured with RA‐FLS and AA‐DHP rat model	Hsa_circ_0091685/EIF4A3/IL‐17	HQC reduces the inflammatory response in RA by suppressing the hsa_circ_0091685/EIF4A3/IL‐17 axis
	HQC	Scutellariae Radix, Coicis Semen, Persicae Semen, Gardeniae Fructus, and Clematidis Radix et Rhizoma	Synovium and FLS of CIA mice	Circ_0015756/miR‐942‐5p/CUL4B/Wnt	HQC interfered with the effects of circ_0015756 on the pathogenesis of RA by inhibiting the CUL4B
Apoptosis	TPL	NA	RA‐PBMcs and RA‐FLS	LncRNA ENST00000619282	The anti‐RA potential of TPL might be achieved by downregulating ENST00000619282 and promoting apoptosis.
	XFC	Astragali Radix, Coicis Semen, Tripterygii Wilfordii Radix, and Scolopendra	RA‐PBMcs and RA‐FLS	lncRNA MAPKAPK5‐AS1	XFC promotes the apoptosis in RA by regulating the expression of lncRNA MAPKAPK5‐AS1
	XFC	Astragali Radix, Coicis Semen, Tripterygii Wilfordii Radix, and Scolopendra	RA‐PBMCs co‐cultured with RA‐FLS	FTO/YTHDF1/ENST00000619282/NF‐κB	XFC promotes RA‐FLS apoptosis by modulating FTO‐mediated m6A modification of ENST00000619282
	XFC	Astragali Radix, Coicis Semen, Tripterygii Wilfordii Radix, and Scolopendra	RA‐FLS	circ‐CBLB	XFC promotes the apoptosis of RA‐FLSs by upregulating the expression of circ‐CBLB
	WTD	Aconiti Radix Cocta, Ephedrae Herba, Paeoniae Radix Alba, Astragali Radix, and Glycyrrhiza Radix Preparata	CIA rat model	Ahr/LOC101928120/SHC1	WTD promotes apoptosis of RA‐FLSs by modulating the AHR/LOC101928120/SHC1 pathway
Oxidative stress	Resveratrol	NA	CIA mice	NRF2/KEAP1	Resveratrol alleviates oxidative stress in CIA mice by activating the NRF2/KEAP1 pathway
	EMO	NA	CIA mice	ROS/TXNIP/NLRP3	EMO ameliorates RA symptoms by attenuating oxidative stress and suppressing inflammasome activation through the ROS/TXNIP/NLRP3 signaling pathway
	Sinomenine (Sino)	NA	CIA mouse model	ROS/NRF2/HO‐1/NQO1	Sino ameliorates RA by enhancing antioxidant capacity through activation of the NRF2/HO‐1/NQO1 signaling pathway.
	CBN	NA	CIA mice	JAK1/STAT3, NF‐κB and Keap1/Nrf2	CBN exert anti‐RA effects from four perspectives: inhibiting inflammatory response, regulating oxidative stress, and improving changes in gut microbiota and metabolites
	XFC	Astragali Radix, Coicis Semen, Tripterygii Wilfordii Radix, and Scolopendra	RA‐FLS and AA rat model	LINC00638/Nrf2/HO‐1	XFC inhibits oxidative stress in RA by up‐regulating LINC00638 and activating Nrf2/HO‐1 pathway
	HQC	Scutellariae Radix, Coicis Semen, Persicae Semen, Gardeniae Fructus, and Clematidis Radix et Rhizoma	RA‐PBMCs	AMPK/FoxO3a	HQC ameliorates oxidative stress in RA by activating AMPK, which directly phosphorylates FOXO3a
Pyroptosis	Stigmasterol (Stig)	NA	Chondrocytes and CIA mouse model	Nrf2/NLRP3	Stig alleviates the progression of RA by reducing chondrocyte damage through the inhibition of NRF2/NLRP3‐mediated pyroptosis.
	Paeoniflorin (MDP)	NA	AA rats and macrophage	TLR4/NLRP3/GSDMD	The therapeutic effect of MDP on AA rats is related to the inhibition of macrophage pyroptosis by regulating the TLR4/NLRP3/GSDMD signaling pathway
	Fisetin and Nicorandil	NA	AA rats	TLR4/NF‐κB/Nrf‐2/HO‐1/OPG/RANKL	Fisetin and Nicorandil ameliorates RA by inhibiting pyroptosis through the modulation of the TLR4/NF‐κB/Nrf‐2/HO‐1/OPG/RANKL signaling axis.
	Ginsenoside Rh2 (Rh2)	NA	AA rat model and macrophages	NLRP3/Caspase11/GSDMD‐N	Rh2 ameliorates RA by inhibiting M1 macrophage polarization and pyroptosis via the NLRP3/Caspase‐1/GSDMD pathway
	Mangiferin and Cinnamic acid	NA	AA mouse and MH7A	TLR4/PI3K/AKT/NFκB/NLRP3	Mangiferin and cinnamic acid ameliorate RRA by interacting with the TLR4/PI3K/AKT/NF‐κB signaling axis to suppress NLRP3 inflammasome activation and modulate pyroptosis
	Kaempferol	NA	RA‐FLS	CASP1‐5/GSDMD/IL‐1β/IL‐18	Kaempferol alleviates RA by binding to CASP1 and inhibiting pyroptosis
	Paeoniflorin (MDP)	NA	RA‐FLS and AA rats	ROS/GRK2/HIF‐1α/NLRP3	MDP alleviates RA by inducing pyroptosis in RA‐FLS via the ROS/GRK2/HIF‐1α/NLRP3 pathway
	PPN	NA	RA‐FLS	NLRP3/Caspase‐1/GSDMD/IL‐1β/IL‐18	PPN inhibits pyroptosis of RA‐FLS through the NLRP3/Caspase‐1/GSDMD signaling pathway
	XFC	Astragali Radix, Coicis Semen, Tripterygii Wilfordii Radix, and Scolopendra	RA‐FLS	NLRP3/GSDMD	XFC alleviates inflammatory response of joints in RA possibly by inhibiting pyroptosis of the FLS through inhibition of the NLRP3/GSDMD pathway
	WBT	Rehmanniae Radix, Rehmanniae Radix Praeparata, Dipsaci Radix, Aconiti Lateralis Radix Praeparata, Angelicae Pubescentis Radix, Drynariae Rhizoma, Cinnamomi Ramulus, Epimedii Folium, Saposhnikoviae Radix, Clematidis Radix et Rhizoma, Gleditsiae Spina, Caprinae Os, Paeoniae Radix Alba, Cibotii Rhizoma, Anemarrhenae Rhizoma, Lycopodii Herba, and Carthami Flos	CIA rats with the syndrome of kidney deficiency	cGAS‐STING	WBT alleviate bone destruction in RA by inhibiting the cGAS‐STING signaling pathway, which leads to the downregulation of pyroptotic proteins and suppression of inflammation
	Bitongqing (BTQ)	Neolitsea cactia (L). Kosterm, Paeonia altaica, Reynoutria japonica Houtt, Atractylodes lancea, Saposhnikovia divaricate, Lonicera japonica Thunb, Gentiana macrophylla Pall, Sinomenium acutum (Thunb). Rehder, Rehmannia glutinosa (Gaertn). DC, Gypsum Fibrosum, Smilax glabra Roxb, and Glycyrrhiza uralensis Fisch. ex DC	CIA rat and macrophage	NLRP3/Caspase‐1/GSDMD	The therapeutic potential of BTQ in CIA lies in its ability to inhibit NLRP3‐mediated macrophage pyroptosis
	Yiqi Yangyin Tongluo methods	Raw astragalus 30 g, Dendrobium 15 g, Polygala 15 g, Achyranthes bidentata 25 g, Honeysuckle 15 g	Chondrocytes	ASIC1a/NLRP3	Yiqi Yangyin Tongluo can attenuate the pyroptosis of RA chondrocytes through the ASIC1a/NLRP3 signaling pathway
Ferroptosis	Panaxynol (PAL)	NA	CIA‐FLS	SLC7A11/GPX4/TNF‐α/IL‐1β	PAL ameliorates RA primarily through the modulation of ferroptosis and the NF‐κB signaling pathway
	AMF	NA	MH7A	PIN1	AMF induced ferroptosis to inhibit the proliferation, migration, invasion and inflammation in RA‐FLS by inhibiting PIN1
	Asiatic acid (AA)	NA	RA‐FLS and CIA model rats	Nrf2‐HMOX1	AA significantly reduced the viability of RA‐FLS and triggered ferroptosis by promoting accumulation of Fe^2+^ via the Nrf2‐HMOX1 pathway, and was effective in relieving inflammation in CIA model rats
	ICA	NA	RA‐FLS	Xc‐/GPX4	ICA protects the cells from death in RA‐FLS via the inhibition of ferroptosis by activating the Xc‐/GPX4 axis
	QRHXD	Atractylodes chinensis, Phellodendron amurense, Smilax glabra Roxb, Lonicera japonica Thunb, Astragalus membranaceus, Paeonia lactiflora Pall, Dioscorea futschauensis Uline ex R. Kunth, Salvia miltiorrhiza Bge, Curcuma phaeocaulis Val, Sinomenium acutum, Polistes olivaceous, and Scolopendra subspinipes mutilans L. Koch	CIA mouse model	FBP1/AMPK	QRHXD attenuate the inflammatory response of RA, and delay bone destruction by inhibiting FBP1 and activating the AMPK signalling pathway
	JFG	Schizonepetae Herba, Saposhnikoviae Radix, Notopterygii Rhizoma et Radix, Angelicae Pubescentis Radix, Bupleuri Radix, Peucedani Radix, Chuanxiong Rhizoma, Aurantii Fructus, Poria, Platycodonis Radix, and Glycyrrhizae Radix et Rhizoma	CIA rat model	AMPK/TLR4/NF‐κB/NLRP3	JFG improved the disturbance of fatty acid metabolism by modulating gut microbiota to activate AMPK, and then inhibited ferroptosis caused by lipid oxidative stress in synovium tissue and prevented RA injury.
Angiogenesis	Clematichinenoside AR (CAR)	NA	RA‐FLS co‐cultured with HUVEC	HIF‐1α/VEGFA/ANG2	CAR exerts its therapeutic effect on RA by suppressing synovial angiogenesis via the HIF‐1α/VEGF/ANG2 axis
	ATO (As2O3)	NA	RA‐FLS co‐cultured with HDMEC	CircHIPK3/miR‐149‐5p/FOXO1/VEGF	ATO suppresses RA‐FLS angiogenesis by downregulating the Circhipk3/miR‐149‐5p/Foxo1/VEGF axis
	GE	NA	AA rat models and HUVEC	SphK1/PI3K/AKt/PFKFB3	GE mproves glycolysis driven angiogenesis in experimentary arthritis by inhibiting SphK1‐PI3K‐Akt‐PFKFB3 signal
	Resveratrol	NA	HUVECs and rat vascular epithelial cells	Rho/ROCK/SIRT1	Resveratrol‐induced SIRT1 activation inhibits glycolysis‐fueled angiogenesis under RA conditions
	XFC	Astragali Radix, Coicis Semen, Tripterygii Wilfordii Radix, and Scolopendra	RA‐FLS co‐cultured with HUVEC	LncRNA HOTAIR/PI3K/AKT	XFC alleviates RA‐FLS‐induced angiogenesis in HUVEC cells by inhibiting the lncRNA HOTAIR/PI3K/AKT pathway
	QRHXD	Atractylodes chinensis, Phellodendron amurense, Smilax glabra Roxb, Lonicera japonica Thunb, Astragalus membranaceus, Paeonia lactiflora Pall, Dioscorea futschauensis Uline ex R. Kunth, Salvia miltiorrhiza Bge, Curcuma phaeocaulis Val, Sinomenium acutum, Polistes olivaceous, and Scolopendra subspinipes mutilans L. Koch	HUVEC	PI3K‐Akt/VEGF‐1/VEGF‐2	QRHXD anti‐angiogenesis of RA might inhibit the PI3K‐Akt signalling pathway and downregulate VEGF‐1 and VEGF‐2.
	EMS	Phellodendri Chinensis Cortex and Atractylodis Rhizoma	MH7A and HUVEC	Wnt/β‐catenin	EMS prevents RA by modulating the Wnt/β‐catenin signaling pathway, thereby inhibiting the aberrant activation of RA‐FLS and angiogenesis
	HT	Salviae Miltiorrhizae Radix et Rhizoma, Dioscoreae Nippponicae Rhizoma, Astragali Radix, Aconiti Lateralis Radix Praeparata, Paeoniae Radix Alba, Corydalis Rhizoma, and Glycyrrhizae Radix et Rhizoma	RA‐FLS co‐cultured with HUVEC	HIF1A‐VEGFA‐ANGPT	HT attenuates RA by inhibiting the HIF1A/VEGFA/ANGPT axis and suppressing angiogenesis
	FHD	Stephaniae Tetrandrae Radix, Glycyrrhizae Radix et Rhizoma, Atractylodis Macrocephalae Rhizoma, and Astragali Radix	MH7A, HUVEC, and CIA rat	HIF‐1α	FHD alleviates RA through regulating HIF‐1α mediated the angiogenesis and the balance between autophagy and apoptosis
Hypercoagulable state	XFC	Astragali Radix, Coicis Semen, Tripterygii Wilfordii Radix, and Scolopendra	RA‐PBMCs co‐cultured with RA‐FLS	LncRNA DSCR9/RPLP2/PI3K/AKT	XFC ameliorates the hypercoagulable state in RA by modulating the lncRNA DSCR9/RPLP2/PI3K/AKT axis
Nets	PHSTF	NA	CIA mouse	Nets	PHSTF can alleviate RA by inhibiting the formation of NETs
	MC	NA	CIA mouse	PADI4/MPO	MC protects the ankle joints against arthritis by inhibiting MPO and PADI4, thereby reducing NET release
	EMO	NA	Neutrophils	IL‐6/TNF‐α	EMO ameliorates RA by promoting neutrophil apoptosis and inhibiting NET formation
	Jianpi Qingre Tongluo prescription (HQC)	Scutellariae Radix, Coicis Semen, Persicae Semen, Gardeniae Fructus, and Clematidis Radix et Rhizoma	Damp‐heat pattern rat model, neutrophils co‐cultured with RA‐FLS	Circ0005732/p38 MAPK	HQC exerts therapeutic effects against RA by modulating circ0005732 to inhibit p38 MAPK signaling pathway‐mediated NET generation
	HQC	Scutellariae Radix, Coicis Semen, Persicae Semen, Gardeniae Fructus, and Clematidis Radix et Rhizoma	PMNs co‐cultured with RA‐FLS	p38 MAPK	HQC can target the p38 MAPK signaling pathway to inhibit NET formation and inflammatory response, thereby ameliorate RA
	SMYAD	Lonicerae Japonicae Flos, Scrophulariae Radix, Angelicae Sinensis Radix, and Glycyrrhizae Radix et Rhizoma	CIA mice and neutrophil	TNF‐α, IL‐1β, IL‐6, CXCL1, CXCL2 and IL‐8	SMYAD effectively restrained arthritis in CIA mice by modulating neutrophil activities
Cellular senescence	Taurine	NA	RA‐FLS	FOXO3	Taurine might be a promising drug against RA by targeting cellular senescence through FOXO3
Gut microbiota	CSO	NA	CIA rat	JAK2‐STAT3	CSO alleviates CIA‐associated muscle atrophy and functional decline by modulating gut microbiota dysbiosis
	Fuzi	NA	CIA‐cold rat	TGR5‐cAMP‐PKA/NLRP3	Fuzi could regulate gut microbiota and microbial bile acid metabolism, the microbial metabolite THDCA acts on TGR5‐cAMP‐PKA signaling pathway and NLRP3 inflammasome to reduce cold‐related arthritis
	CBD	NA	CIA rat, neutrophils, macrophages and RA‐FLS	L‐carnitine and butyric acid	CBD regulates L‐carnitine and butyric acid metabolism by modulating the gut microbiota to ameliorate CIA
	WTD	Aconiti Radix Cocta, Ephedrae Herba, Paeoniae Radix Alba, Astragali Radix, and Glycyrrhiza Radix Preparata	AIA rat	Bacteroides, Prevotella, Akkermansia and their associated acetic acid, butyric acid, cholic acid and indole propionic acid	The anti‐RA effects of WTD were partially mediated by gut microbiota and microbial metabolites
	DSD	Angelica sinensis, Cinnamomi cassia, Paeonia lactiiflora, Tetrapanax papyriferus, Asarum heterotropoides, Glycyrrhiza uralensis andZiziphus jujuba Mill	CIA rat	Taurine, urea, betaine, pyruvate, hippurate, succinate, and aceton	DSD exerts its therapeutic effects on RA by ameliorating intestinal metabolites in CIA
Gut microbiota	ZBD	Curculigo orchioides Gaertn, Epimedium brevicornu Maxim, Morinda officinalis How, Angelica sinensis Diels, Anemarrhena asphodeloides Bunge, Cortex Phellodendri Chinsis, Scorpio, and Centipede	CIA rat	Ligilactobacillus, Prevotella_9, unclassified_Bacilli, and unclassified_rumen_bacterium_JW32	ZBD exerts anti‐RA effects by modulating host metabolism and gut flora, as well as regulating inflammatory and immune‐related signaling pathways
	ZT	Coumarins and flavonoids	CIA rat	Propionate, butyrate, and valerate	ZT could regulate the levels of propionate, butyrate, and valerate in CIA rats
	Bitongling (NBTL)	Cinnamomi ramulus, Saposhnikoviae radix, Ephedrae herba, Sinomenii caulis, Aconiti radix, and Vespae nidus	CIA rat	VEGF/VEGFR1/VEGFR2/HIF‐1α	NBTL exhibits significant therapeutic potential in RA by modulating gut microbiota and inhibiting the VEGF signaling pathway
	DZGP	Euphorbiae Ebrateolatae Radix, Araliae Chinensis Radix, Acanthopanacis Senticosi Radix et Rhizoma seu Caulis, and Notoginseng Radix et Rhizoma	CIA mice	NF‐κB	DZGP offers an advantage in RA by changing the metabolic profile, gut microbiota while exhibiting lower cellular toxicity

**FIGURE 3:**
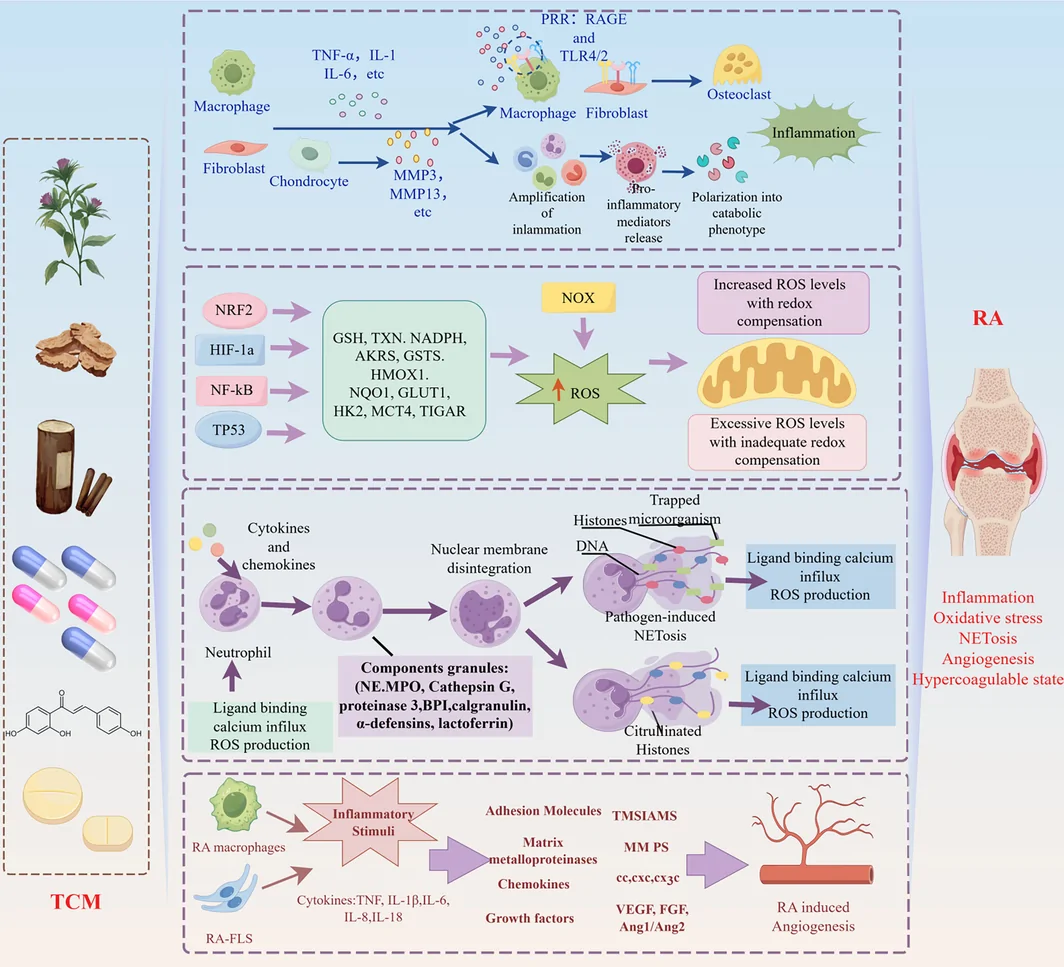
Multi‐targeted therapeutic mechanisms of TCM in RA: Modulation of inflammation, oxidative stress, angiogenesis, and NETs. TCM interventions alleviate RA through coordinated regulation of multiple pathogenic processes. Specifically, in the inflammatory axis, TCM suppresses pro‐inflammatory cytokines, matrix‐degrading enzymes, fibroblast activation, osteoclastogenesis, and amplification of inflammatory mediators. In oxidative stress, TCM modulates redox‐sensitive pathways (including NRF2‐, HIF‐1α‐, NF‐κB‐, and TP53‐related signalings), thereby restoring antioxidant defenses, reducing excessive ROS, and improving mitochondrial redox balance. Moreover, TCM also inhibits NETosis by reducing calcium influx, ROS production, nuclear membrane disintegration, and the release of neutrophil‐derived inflammatory components. Additionally, TCM attenuates angiogenesis by suppressing inflammatory stimuli, adhesion molecules, MMPs, chemokines, and pro‐angiogenic factors (such as VEGF, FGF, and Ang1/Ang2). Through these integrated actions, TCM counteracts key pathological events in RA, including inflammation, oxidative stress, NETosis, angiogenesis, and hypercoagulability.

**FIGURE 4:**
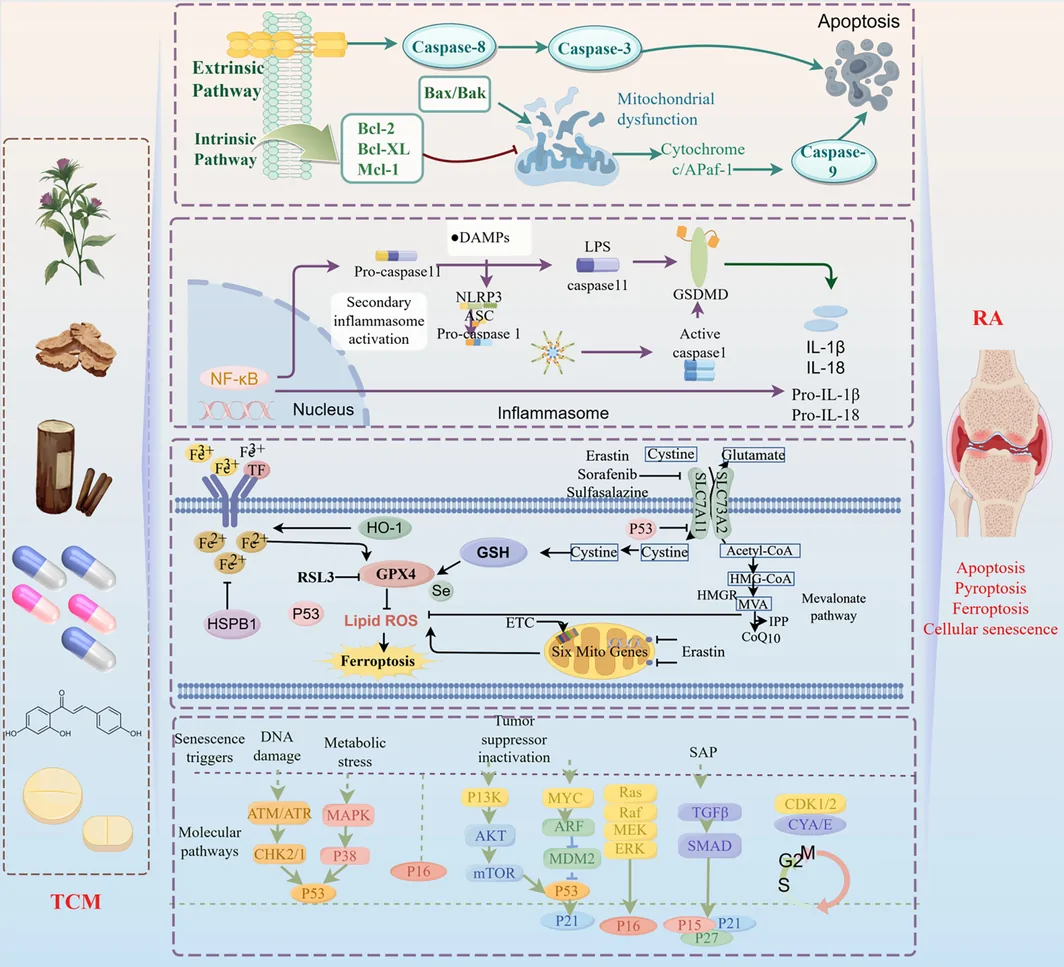
Integrated view of cell death pathways in RA: From apoptosis and pyroptosis to ferroptosis and senescence. TCM interventions alleviate RA by modulating multiple forms of regulated cell death and senescence‐associated pathways. In apoptosis, TCM regulates Bcl‐2 family proteins, mitochondrial dysfunction, cytochrome c release, Apaf‐1 activation, and downstream caspase signaling, thereby influencing both extrinsic and intrinsic pathways. In pyroptosis, TCM suppresses inflammasome activation and the cleavage of caspase‐1/caspase‐11 and GSDMD, thereby reducing the maturation and release of IL‐1β and IL‐18. In ferroptosis, TCM targets iron metabolism, lipid peroxidation, GSH/GPX4‐dependent antioxidant defense, cystine transport, and mitochondrial metabolic pathways, thereby regulating ferroptotic cell death. In cellular senescence, TCM modulates multiple senescence‐associated signaling networks (including ATM/ATR–CHK2–p53, MAPK, PI3K/AKT/mTOR, ARF/MDM2, TGFβ/SMAD, and CDK/cyclin‐related pathways), thereby influencing cell‐cycle arrest and senescence‐associated phenotypes. Taken together, through these integrated actions, TCM counteracts key pathogenic processes contributing to RA progression.

**FIGURE 5:**
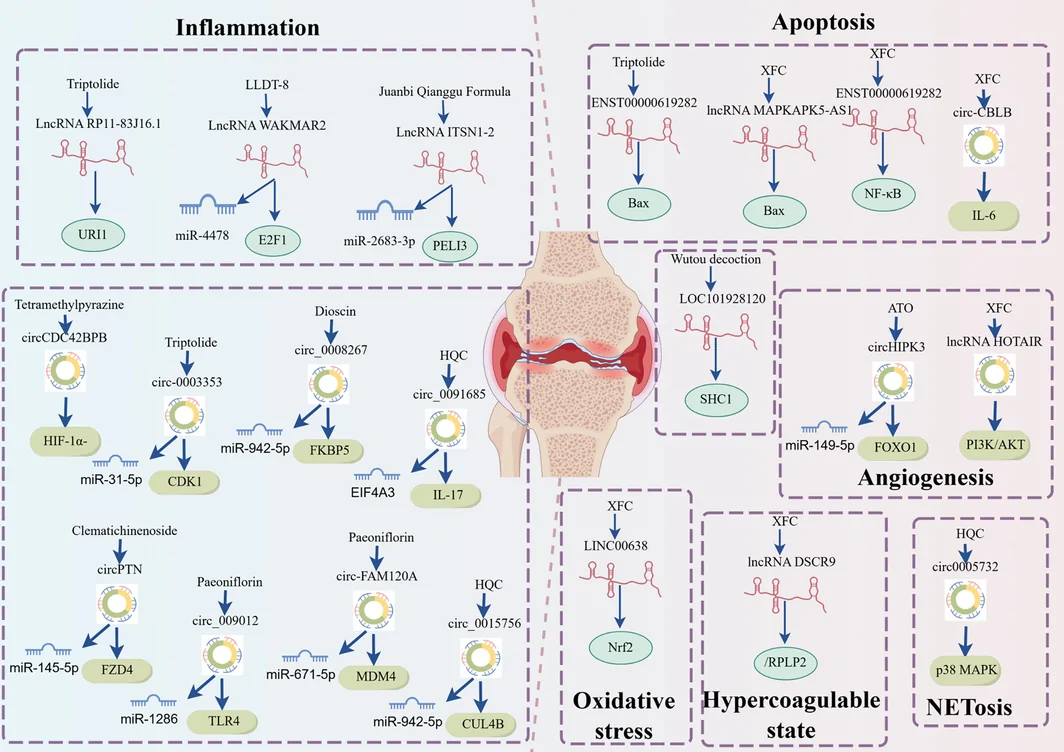
TCM regulation of the ceRNA network in the treatment of RA. The figure summarizes selected lncRNA‐ and circRNA‐associated molecular axes involved in the therapeutic effects of TCM on RA across multiple pathogenic processes. In inflammation, TCM monomers and formulas modulate lncRNA/circRNA‐centered networks to regulate key inflammatory mediators and signaling molecules, including URI1, E2F1, PELI3, HIF‐1α, CDK1, FKBP5, EIF4A3, IL‐17, FZD4, TLR4, MDM4, and CUL4B. In apoptosis, TCM interventions influence apoptosis‐related pathways through ncRNAs linked to Bax, TNF‐κB, IL‐6, and SHC1. In angiogenesis, circHIPK3‐ and HOTAIR‐associated axes regulate FOXO1 and PI3K/AKT signaling. In addition, ncRNA‐mediated regulation is involved in oxidative stress, hypercoagulability, and NETosis through representative targets (such as Nrf2, RPLP2, and p38 MAPK). Taken together, these findings illustrate that distinct TCM interventions converge on multiple ncRNA‐regulated networks, which underlie the multi‐target therapeutic actions of TCM in RA.

### 5.1 Inflammation

TCM has been demonstrated to exert therapeutic effects on RA through multi‐target modulation of inflammatory signaling pathways and suppression of key inflammatory mediators. Notably, TCM modulates ncRNAs, which are critically involved in post‐transcriptional regulation (Wen et al. 2023). Different TCM monomers and formulas appear to converge on several common inflammatory hubs (including NF‐κB, MAPK, and inflammasome‐related pathways), rather than acting through isolated molecular events, thereby reducing the production and release of cytokines (e.g., TNF‐α, IL‐1β, and IL‐6). This broad suppression of inflammatory cascades contributes to alleviating synovitis, preventing cartilage destruction, and attenuating bone erosion.

### 5.2 LncRNA

#### 5.2.1 TCM Monomer

Several TCM monomers suppress RA‐associated inflammation through lncRNA‐mediated regulation of shared downstream pathways. Triptolid is a TCM compound widely used in RA treatment in China. It has been shown to markedly inhibit RA‐FLS proliferation, invasion, and pro‐inflammatory cytokine secretion, while also enhancing apoptosis (Wang, Liu, et al. 2025; Wang, Ni, et al. 2025; Wang, Pan, et al. 2025; Wang, Wen, et al. 2025; Wang, Zhang, et al. 2025; Piao et al. 2021). Mechanistically, these effects are associated with downregulation of lncRNA RP11‐83J16.1 and inhibition of the URI1/β‐catenin signaling. It has been shown that TPL similarly attenuates arthritis severity, synovial hyperplasia, inflammatory infiltration, and cytokine levels in collagen‐induced arthritis (CIA) rats, and these effects are reversed by lncRNA RP11‐83J16.1 overexpression (Piao et al. 2021). Likewise, the TPL derivative (5R)‐5‐hydroxytriptolide (LLDT‐8) significantly suppresses pro‐inflammatory cytokine production in RA‐FLS by upregulating lncRNA WAKMAR2, which functions through the miR‐4478/E2F1/p53 axis (Zhou et al. 2021). Taken together, these findings suggest that structurally related TCM monomers may regulate different lncRNA‐centered networks while leading to common inflammatory outcomes.

#### 5.2.2 TCM Compound

TCM formulas also exert anti‐inflammatory effects through lncRNA‐associated mechanisms. For example, Juanbi Qianggu Formula (JBQG) is a clinically effective TCM prescription, which has been shown to remarkably suppress inflammatory responses and delay RA progression by targeting the lncRNA ITSN1‐2/miR‐2683‐3p/PELI3/RIP2 axis, thereby inhibiting the NF‐κB signaling in RA‐FLS (Bian et al. 2025). Additionally, another representative classical TCM formula Huo‐Luo‐Xiao‐Ling Dan (HLXL) has also been extensively validated in preclinical models of RA, particularly adjuvant arthritis. Accumulating studies have indicated that HLXL exerts anti‐inflammatory and joint‐protective effects through multiple mechanisms. Specifically, it modulates antigen‐induced immunological and biochemical mediators of inflammation, suppresses chemokine production and matrix‐degrading enzymes, and attenuates bone damage by regulating bone remodeling‐related mediators (Yang et al. 2011; Nanjundaiah et al. 2012; Nanjundaiah et al. 2013). These findings further support the multi‐target therapeutic potential of classical herbal formulas in RA.

Collectively, these findings highlight the therapeutic potential of TCM compounds and TCM formulas in suppressing RA‐associated inflammation. This effect is achieved through the regulation of inflammatory mediators, ncRNA‐associated pathways, and tissue‐destructive processes.

### 5.3 CircRNA

#### 5.3.1 TCM Monomer

CircRNAs, a class of endogenous ncRNAs characterized by covalently closed loop structures, have emerged as pivotal regulators of post‐transcriptional gene expression; they provide additional insight into how TCM interventions modulate RA inflammation (Tang et al. 2025). CircRNAs commonly function as ceRNAs by sequestering miRNAs through miRNA response elements (MREs), thereby regulating target genes involved in RA pathological processes. Several TCM monomers have been shown to modulate distinct circRNA‐mediated axes. For example, TMP, an alkaloid monomer isolated from *Ligusticum wallichii* Franch., has been shown to suppress inflammation in RA‐FLS by downregulating hypoxia‐induced circCDC42BPB, thereby attenuating FLS dysfunction (Zhang, Chen, et al. 2024; Zhang, Weng, et al. 2024). TPL also inhibits RA‐FLS proliferation and cytokine production through the circ0003353/miR‐31–5p/CDK1 axis, which links circRNA regulation to cell‐cycle control and inflammatory progression (Wen et al. 2022). Additionally, it has been shown that dioscin suppresses RA‐FLS proliferation, invasion, migration, and inflammatory responses through the circ_0008267/miR‐942‐5p/FKBP5 axis (Chen, Guo, Wu, et al. 2025; Chen, Xu, Chen, et al. 2025; Chen, Xu, Zhang, and Xia 2025). Similarly, *Clematis Chinensis* Osbeck has been reported to attenuate RA progression via regulating the circPTN/miR‐145‐5p/FZD4 signaling axis (Wang, Wang, et al. 2022; Wang, Zhou, et al. 2022). PF, the major active component of total paeony glycosides, exerts anti‐inflammatory effects by downregulating hsa_circ_009012, thereby inhibiting the TLR4/NLRP3 inflammasome axis via miR‐1286. It also suppresses circ‐FAM120A to elevate miR‐671–5p and inhibit MDM4, ultimately reducing RA‐FLS proliferation, migration, and inflammation (Ma et al. 2021; Yang, Shu, et al. 2024; Yang, Wei, et al. 2024). Taken together, these studies indicate that different TCM monomers may regulate distinct circRNA‐mediated ceRNA networks but lead to similar anti‐inflammatory and tissue‐protective effects.

#### 5.3.2 TCM Compound

TCM formulas also regulate circRNA‐mediated inflammatory signaling in RA. For example, HQC is a prescription used by the First Affiliated Hospital of Anhui University of Traditional Chinese Medicine and approved by the Anhui Provincial Medical Products Administration (approval no. Z20200001). It has been shown to markedly reduce inflammatory responses in RA patients (Hu et al. 2024). HQC is composed of *Scutellariae Radix* (Huangqin), *Persicae Semen* (Taoren), *Clematidis Radix* (Weilingxian), *Coicis Semen* (Yiyiren), and *Gardeniae Fructus* (Zhizi), which is traditionally used for dampness‐heat syndrome rheumatic diseases. As revealed by mechanistic investigations, HQC exerts anti‐arthritic and anti‐inflammatory effects by inhibiting the hsa_circ_0091685/EIF4A3/IL‐17 axis, thereby suppressing key pro‐inflammatory pathways involving IL‐17, TNF‐α, IL‐6, and IL‐23 (Hu et al. 2024). Additional evidence further shows that HQC inhibits inflammation in RA by targeting CUL4B and suppressing the Wnt signaling via modulating the circ_0015756/miR‐942‐5p/CUL4B/Wnt cascade (Wang, Chang, et al. 2021; Wang, Chen, et al. 2021). These findings support that TCM formulas can regulate multiple inflammatory nodes through distinct circRNA‐associated pathways. From the perspective of TCM theory, the therapeutic effects of TCM formulas on RA may involve both clearing heat and removing toxins, as well as resolving stasis and restoring local tissue balance.

Collectively, TCM suppresses RA inflammation not by inhibiting isolated cytokines or single ncRNA axes. Instead, different TCM monomers and formulas act through diverse lncRNA and circRNA pathways to converge on common inflammatory hubs and regulate a broader pathogenic network. Moreover, inflammation in RA is closely interconnected with oxidative stress, pyroptosis, ferroptosis, angiogenesis, and cellular senescence. Excessive inflammatory signaling amplifies ROS production and tissue hypoxia, which in turn further activate NF‐κB‐related cascades, inflammasome signaling, lipid peroxidation, and SASP. Therefore, the anti‐inflammatory effects of TCM may have broader regulatory significance. They not only directly suppress inflammatory mediators but also modulate a wider pathological network that links immune dysregulation to oxidative and degenerative damage in RA.

### 5.4 Apoptosis

TCM exerts therapeutic effects on RA partly through modulating key apoptotic signaling pathways, particularly via crosstalk with ncRNAs, thereby promoting cell apoptosis and ameliorating disease progression. Restoring apoptotic balance helps inhibit synovial hyperplasia and joint destruction, which are hallmark pathological features of RA.

#### 5.4.1 TCM Monomer

A previous study investigating the mechanism of TPL in RA has identified lncRNA ENST00000619282 as a key regulatory molecule related to apoptosis (Wen et al. 2021a, 2021b). Specifically, this lncRNA is significantly upregulated in RA‐peripheral blood mononuclear cells (PBMCs) and is positively correlated with disease activity markers. According to in vitro experiments, ENST00000619282 overexpression promotes pro‐inflammatory responses and inhibits FLS apoptosis. Critically, TPL treatment could effectively downregulate ENST00000619282, thereby inducing apoptosis in RA‐FLS. These findings suggest that TPL exerts its anti‐RA effects, at least in part, through negative regulation of lncRNA ENST00000619282.

#### 5.4.2 TCM Compound

XFC is composed of *Astragali Radix* (Huangqi), *Coicis Semen* (Yiyiren), *Tripterygium wilfordii* (Leigongteng), and *Scolopendra* (Wugong) in optimized ratios. It exhibits spleen‐strengthening, dampness‐resolving, and collateral‐dredging properties. Moreover, XFC has been approved by the Anhui Provincial Medical Product Administration (approval no.: Z20050062). Wen et al. (2021a, 2021b) have reported that XFC alleviates RA by upregulating lncRNA MAPKAPK5‐AS1, which is significantly decreased in RA patients. Through this mechanism, XFC promotes RA‐FLS apoptosis and reduces inflammatory responses, as evidenced by altered expression of IL‐17, IL‐4, Bax, and Bcl‐2. Additionally, it has been shown that XFC inhibits the demethylase FTO, thereby increasing m6A RNA methylation of lncRNA ENST00000619282. This modification reduces the lncRNA's stability and YTHDF1‐mediated expression, and ultimately suppresses the PUF60/NF‐κB pathway, thereby promoting FLS apoptosis and attenuating inflammation (Wang, Liu, et al. 2025; Wang, Ni, et al. 2025; Wang, Pan, et al. 2025; Wang, Wen, et al. 2025; Wang, Zhang, et al. 2025). Moreover, XFC‐containing serum has also been shown to upregulate circRNA CBLB expression in TNF‐α‐stimulated RA‐FLS, resulting in cell‐cycle arrest and enhanced apoptosis (Li, Liu, et al. 2024; Li, Wan, et al. 2024). WTD, derived from Zhang Zhongjing (a famous Chinese medicine practitioner), is composed of 5 TCM herbs (*Radix Aconiti*, *Herba Ephedra*, *Radix Astragali*, *Radix Paeoniae Alba*, and *Radix Glycytthizae*); it has been widely used for RA treatment in China. Wu et al. (2021) have demonstrated that WTD activates the Ahr transcription factor, which upregulates lncRNA LOC101928120. This lncRNA then recruits histone deacetylase HDAC1 to the promoter of the SHC1 gene, repressing SHC1 expression. Downregulation of the SHC1 oncoprotein subsequently attenuates chondrocyte apoptosis, revealing a novel Ahr/lncRNA/HDAC1 axis for RA treatment.

Collectively, these findings indicate that different TCM monomers and formulas restore apoptotic balance in RA through distinct ncRNA and epigenetic pathways, yet they all converge on promoting RA‐FLS apoptosis and suppressing inflammatory progression.

### 5.5 Oxidative Stress

TCM has garnered increasing attention for its potential to ameliorate RA by targeting oxidative stress pathways. In general, different TCM monomers and formulas act through distinct pathways but converge on restoring redox homeostasis by activating antioxidant pathways, particularly the Nrf2‐related axis. This reduces ROS accumulation, limits oxidative damage, and suppresses inflammation‐driven tissue injury.

#### 5.5.1 TCM Monomer

Representative studies have shown that several TCM‐derived compounds alleviate RA‐associated oxidative stress through overlapping antioxidant mechanisms. For example, resveratrol has been shown to attenuate hepatic inflammation and oxidative stress in CIA mice and TNF‐α/H_2_O_2_‐stimulated primary hepatocytes by activating the Nrf2/Keap1 pathway; mechanistic intervention with Nrf2 inhibition or Keap1 overexpression further confirms the central role of this axis (Fan et al. 2025). Similarly, it has been shown that EMO ameliorates RA by reducing ROS and malondialdehyde (MDA) levels, increasing SOD activity, and inhibiting the TXNIP/NLRP3 inflammasome pathway in CIA mice and LPS‐stimulated macrophages (Zhou, Liu, et al. 2025; Zhou, Wu, et al. 2025; Zhou, Yang, et al. 2025). Additionally, SINX, a sinomenine derivative, has also been reported to alleviate bone destruction by improving mitochondrial dysfunction, reducing ROS, and activating the Nrf2/HO‐1/NQO1 antioxidant pathway, thereby suppressing osteoclastogenesis and bone resorption (Guo et al. 2025). Moreover, columbianadin (CBN) alleviates arthritis by activating the Keap1/Nrf2 pathway, rebalancing redox homeostasis, and modulating gut microbiota‐associated metabolites (Chen, Wang, Wang, et al. 2023; Chen, Wang, Zhang, et al. 2023).

#### 5.5.2 TCM Compound

TCM formulas also regulate oxidative stress through both canonical antioxidant pathways and ncRNA‐mediated mechanisms. For instance, XFC upregulates LINC00638, activates the Nrf2/HO‐1 pathway, and reduces IL‐6, IL‐17, ROS, and RNS levels in RA‐FLS, thereby alleviating RA (Sun et al. 2023) In addition, HQC‐containing serum exerts significant antioxidative and anti‐inflammatory effects via activating the AMPK/FoxO3a pathway. This leads to increased TAOC and SOD levels, reduced MDA and lipid peroxide (LPO) levels, suppressed IL‐1β, and promoted IL‐4 expression; these effects were attenuated by AMPK inhibition (Guo et al. 2020).

Collectively, these findings suggest that TCM‐mediated regulation of oxidative stress is not limited to simple ROS scavenging. Rather, it involves coordinated modulation of redox‐sensitive signaling, inflammatory pathways, and metabolic homeostasis, thereby contributing to broader disease control in RA.

### 5.6 Pyroptosis

Emerging evidence suggests that TCM can effectively regulate RA‐associated pyroptosis through multi‐component and multi‐target mechanisms. Although the specific upstream triggers vary across studies, most currently available evidence shows that TCM consistently suppresses inflammasome activation, inhibits caspase‐1/GSDMD signaling, and reduces IL‐1β and IL‐18 release.

#### 5.6.1 TCM Monomer

##### 5.6.1.1 Animal Experiments

Representative in vivo studies have indicated that PF‐derived and phytochemical interventions can suppress pyroptosis‐related injury in RA. For instance, the PF derivative muramyl dipeptide (MDP) ameliorates adjuvant arthritis in rats by suppressing macrophage pyroptosis via inhibiting the TLR4/MyD88/NLRP3/Caspase‐1/GSDMD‐N axis, accompanied by reduced IL‐1β and IL‐18 levels and restored M1/M2 macrophage balance (Xu et al. 2021). Additionally, stigmasterol has been evidenced to attenuate chondrocyte injury by activating the Nrf2 signaling and suppressing NLRP3 inflammasome‐mediated pyroptosis (Ding et al. 2025). Other interventions have also been reported to attenuate RA‐associated pyroptosis through suppression of inflammasome‐related signaling pathways. These interventions include fisetin combined with nicorandil, mesenchymal stem cell exosomes (MSC‐exo) plus ginsenoside Rh2, and Baihu‐Guizhi decoction (Li et al. 2022; Okasha et al. 2025; Zhou, Liu, et al. 2025; Zhou, Wu, et al. 2025; Zhou, Yang, et al. 2025).

##### 5.6.1.2 Cell Experiments

At the cellular level, kaempferol directly targets pyroptosis in RA‐FLS; it shows strong binding to CASP1 and reduces the expression of CASP1, GSDMD, GSDMD N‐terminal domain (GSDMD‐NT), IL‐1β, and IL‐18, thereby attenuating pyroptotic cell death (Ling et al. 2024). Hypoxia‐induced FLS pyroptosis is mediated through the ROS/GRK2/HIF‐1α/NLRP3 pathway; MDP suppressed this process via inhibiting GRK2 phosphorylation (Hong et al. 2022). Similarly, periplogenin (PPN) has been shown to reduce RA‐FLS growth, migration, invasion, and IL‐1β/IL‐18 secretion by inhibiting the NLRP3/Caspase‐1/GSDMD pathway (Ma et al. 2024).

#### 5.6.2 TCM Compound

TCM formulas also exert robust anti‐pyroptotic effects on RA. As has been evidenced previously, XFC‐containing serum significantly alleviates LPS‐induced pyroptosis in RA‐FLS by reducing pyroptotic morphology, IL‐1β and IL‐18 release, and expression of NLRP3, caspase‐1, and GSDMD, showing comparable effects to those of MCC950 (Wang, Wang, et al. 2022; Wang, Zhou, et al. 2022). Wangbi tablets (WBT) attenuate joint pathology and reduce IL‐18 and IL‐1β levels in CIA rats by upregulating Polβ and inhibiting the cGAS‐STING pathway, thereby downregulating GSDMD, GSDMD‐NT, and caspase‐1 (Bian et al. 2024). Additionally, Bitongqing also ameliorates RA progression by suppressing pyroptosis‐related pathways, based on the strategy of “nourishing qi, nourishing yin, and dredging collaterals” (Wu et al. 2024; Zhao, Chen, et al. 2022; Zhao, Wei, et al. 2022; Zhao, Yang, et al. 2022).

Importantly, pyroptosis is mechanistically linked to multiple pathological pathways in RA rather than functioning as an independent process. Oxidative stress promotes inflammasome activation, while pyroptotic release of IL‐1β and IL‐18 further amplifies inflammatory cascades and aggravates synovial injury. Moreover, persistent pyroptotic signaling may cooperate with mitochondrial dysfunction, lipid peroxidation, and senescence‐associated inflammatory responses, thereby forming a feed‐forward loop that accelerates joint destruction. From this perspective, TCM‐mediated inhibition of pyroptosis may have broader therapeutic significance, as it can simultaneously attenuate inflammation, oxidative injury, and downstream tissue remodeling.

### 5.7 Ferroptosis

More direct evidence suggests that active components from TCM formulations can target key regulators of ferroptosis, such as GPX4, System Xc‐, and Nrf2, thereby restoring redox homeostasis and mitigating RA‐FLS death. This presents a promising multi‐component, multi‐target therapeutic strategy for RA treatment.

#### 5.7.1 TCM Monomer

Representative studies have shown that several TCM monomers use different upstream mechanisms to regulate ferroptosis, but all target lipid peroxidation, iron metabolism, and inflammatory control. For example, amentoflavone (AMF), a polyphenol from *Selaginella tamariscina*, directly binds to and inhibits PIN1, thereby promoting ferroptosis and suppressing RA‐FLS proliferation, migration, and inflammation (Ma et al. 2025). In contrast, ICA is a flavonoid from *Epimedium*; it inhibits ferroptosis in LPS‐induced RA‐FLS by activating the Xc‐/GPX4 antioxidant axis and upregulating Nrf2, thereby reducing lipid peroxidation and iron accumulation (Luo and Zhang 2021). Additionally, other natural compounds (such as PAL and AA) have also been reported to regulate ferroptosis‐related pathways in RA‐FLS through ceRNA networks or Nrf2‐HMOX1 signaling (Sun et al. 2024; Sun, Liu, He, et al. 2025; Sun, Liu, Wang, et al. 2025).

#### 5.7.2 TCM Compound

Qingre Huoxue Decoction (QRHXD), a TCM formula widely used in clinical practice, has exhibited significant therapeutic effects on RA. Recent studies have indicated that QRHXD could effectively improve clinical scores [Disease Activity Score in 28 joints (DAS28), CRP, and ESR] and modulate key pathways involving ferroptosis and lipid metabolism. According to integrated proteomic and metabolomic analyses, QRHXD regulates the AMPK signaling and suppresses fructose‐1,6‐biphosphatase 1 (FBP1), thereby attenuating inflammation and delaying bone destruction (Zhang et al. 2025). Similarly, Jingfang Granule (JFG), another TCM formula, has been shown to ameliorate RA by modulating the gut‐joint axis. Specifically, JFG restores gut microbiota diversity and increases fecal and serum short‐chain fatty acids (SCFAs). These SCFAs inhibit NLRP3‐ and TLR4/NF‐κB‐mediated inflammation and suppress lipid oxidative stress‐induced ferroptosis in synovial tissue. JFG also strengthens the intestinal barrier (Wang, Liu, et al. 2025; Wang, Ni, et al. 2025; Wang, Pan, et al. 2025; Wang, Wen, et al. 2025; Wang, Zhang, et al. 2025).

Notably, oxidative stress, cellular senescence, and ferroptosis are closely interrelated in RA progression. Specifically, oxidative stress serves as a crucial upstream trigger for lipid peroxidation and ferroptotic cell death, while chronic redox imbalance also drives premature senescence of synovial and immune cells. Senescent cells further sustain inflammatory signaling and oxidative damage through the SASP, thereby reinforcing a vicious cycle. This interconnected relationship suggests that by regulating redox homeostasis, TCM may simultaneously influence ferroptosis sensitivity and senescence‐associated inflammation, revealing a systems‐level mechanism underlying its therapeutic effects in RA.

### 5.8 Angiogenesis

More evidence highlights that TCM exerts therapeutic effects on RA by modulating angiogenesis, a key process in synovial pannus formation. Through multi‐target mechanisms, active compounds and formulas regulate pro‐ and anti‐angiogenic factors, notably suppressing VEGF, FGF, and angiopoietin pathways. Simultaneously, TCM mitigates inflammation and immune dysregulation, disrupting the vicious cycle between angiogenesis and synovitis. This holistic approach suppresses abnormal vascular proliferation while also improving microcirculation and tissue repair.

#### 5.8.1 TCM Monomer

Representative monomers have demonstrated clear anti‐angiogenic activity in RA‐related models. It has been shown that clematichinenoside AR suppresses synovial angiogenesis by targeting HIF‐1α and downregulating the VEGF/ANG2 signaling axis (Yuan et al. 2025). Additionally, geniposide (GE) inhibits endothelial metabolic reprogramming by suppressing the SphK1‐PI3K‐Akt/PFKFB3 pathway, thereby reducing endothelial tube formation and synovial vascularization (Bu et al. 2025). Moreover, resveratrol‐mediated SIRT1 activation inhibits endothelial glycolysis and downstream Rho/ROCK signaling, thereby downregulating VEGF expression and impairing endothelial migration and tube formation (Jiang, Ji, et al. 2023; Jiang, Wang, et al. 2023). Arsenic trioxide (ATO) has also been shown to suppress synovial angiogenesis via regulating a circHIPK3/miR‐149‐5p/FOXO1 module (Zhang, Lei, and Li 2021; Zhang, Ma, et al. 2021).

#### 5.8.2 TCM Compound

TCM formulas also exert anti‐angiogenic effects through multi‐target mechanisms. For instance, a previous study has revealed that XFC serum downregulates the lncRNA HOTAIR/miR‐126‐3p axis in human umbilical vein endothelial cells (HUVECs) co‐cultured with RA‐FLS. This suppresses PI3K/AKT activation and reduces VEGF and bFGF expression, thereby inhibiting angiogenesis (Liu, Qu, et al. 2024; Liu, Wang, et al. 2024). Er Miao San (EMS), a classical TCM formula for damp‐heat syndrome, has been demonstrated to ameliorate RA by inhibiting synovial angiogenesis and FLS activation. Mechanistically, in both adjuvant arthritis rats and MH7A cells, EMS suppresses the Wnt/β‐catenin signaling pathway, reduces pro‐angiogenic factors, and decreases blood flow and immature blood vessel formation (Chen, Wang, Wang, et al. 2023; Chen, Wang, Zhang, et al. 2023). From an ethnopharmacological perspective, these anti‐angiogenic and microenvironment‐modulating effects may reflect the traditional actions of clearing “heat” and resolving “stasis”. Abnormal vascular proliferation, local hyperemia, and persistent synovial activation are biomedical correlates of these pathological states in RA. Additionally, Huayu Tongbi formula (HT) attenuates RA‐associated angiogenesis by suppressing the HIF1A‐VEGFA‐ANGPT axis and modulating proline and tyrosine metabolism, thereby disrupting RA‐FLS/HUVEC crosstalk (Chen, Guo, Wu, et al. 2025; Chen, Xu, Chen, et al. 2025; Chen, Xu, Zhang, and Xia 2025). QRHXD and Fangji Huangqi Decoction (FHD) have also been shown to suppress synovial angiogenesis via regulating PI3K/Akt‐ or HIF‐1α‐related pathways (Zhang, Wang, et al. 2023; Zhang, Zhi, et al. 2023; Zhang, Zhu, and Wu 2023; Xu, Li, Liu, et al. 2024; Xu, Li, Wang, et al. 2024).

Collectively, these studies indicate that TCM limits synovial angiogenesis not only by directly suppressing pro‐angiogenic mediators but also by remodeling the inflammatory and metabolic microenvironment that supports pannus formation.

### 5.9 Hypercoagulable State

XFC exerts potent dual anti‐coagulant and anti‐inflammatory effects in RA treatment. However, its underlying mechanisms require further elucidation. A recent study has revealed that XFC ameliorates inflammatory and hypercoagulable states in RA by modulating the lncDSCR9/RPLP2/PI3K/AKT axis (Wang, Fu, et al. 2024; Wang, Kong, and Li 2024; Wang and Liu 2024; Wang, Liu, et al. 2024; Wang, Xu, et al. 2024). Clinical assessments reveal correlations between XFC treatment and improvements in inflammatory markers, coagulation parameters, and quality of life. Integrative approaches (including network pharmacology and functional experiments) have revealed that XFC upregulates lncRNA DSCR9, which in turn suppresses PI3K/AKT activation. Both in vitro and in vivo studies confirm that XFC reduces inflammation and hypercoagulability, and these effects are reversible by a PI3K/AKT agonist. These findings highlight the role of XFC in targeting the lncDSCR9‐mediated PI3K/AKT pathway, providing a mechanistic basis for its anticoagulant therapeutic potential in RA. Taken together, these results support that TCM can target the inflammation‐coagulation interface through integrated molecular regulation.

### 5.10 NETs

New evidence suggests that TCM can effectively regulate the formation and degradation of NETs, thereby altering RA progression (Liu, Qu, et al. 2024; Liu, Wang, et al. 2024). Active ingredients from TCM compounds have been shown to inhibit NETosis by suppressing ROS generation, peptidyl arginine deiminase 4 (PAD4) activity, and myeloperoxidase (MPO) expression. Furthermore, TCM enhances the clearance of NETs by modulating phagocytic function and mitigating subsequent inflammatory cascades. This regulatory effect on NETs not only reduces joint injury but also helps restore immune homeostasis. Although upstream interventions vary, many TCM‐derived compounds and formulas converge on suppressing ROS production, PAD4 activity, MPO function, and downstream NET‐driven inflammatory crosstalk.

#### 5.10.1 TCM Monomer

Yang, Shu, et al. (2024) and Yang, Wei, et al. (2024) have proved that *Pterocardia hupehensis* Skan total flavonoids (PHSTF), a phytomedicine, significantly alleviates RA by targeting NETs. Specifically, in a CIA rat model, PHSTF treatment reduces ankle swelling, downregulates pro‐inflammatory cytokines, and suppresses the expression of the NET marker Cit‐H3 in vivo. Mechanistically, PHSTF directly inhibits PMA‐induced NETosis by downregulating key mediators such as PADI4, NE, and MPO. These findings provide compelling evidence that PHSTF exerts its therapeutic effect by mitigating NET formation, highlighting a novel mechanism for herbal interventions in RA. Additionally, myricetin (MC), a natural flavonoid, also demonstrates notable anti‐arthritic effects. It has been shown that MC alleviates RA by inhibiting NET release, largely through blocking the nuclear translocation of PADI4 and MPO and preventing subsequent chromatin decondensation (Shu et al. 2024). EMO has also been reported to alleviate joint inflammation in adjuvant‐induced arthritis by promoting neutrophil apoptosis while suppressing autophagy and NETosis (Zhu et al. 2019).

#### 5.10.2 TCM Compound

Representative formulas also regulate NET‐driven pathology through multi‐target mechanisms. For example, HQC alleviates synovial pathology and systemic inflammation by targeting a circ0005732/p38 MAPK axis and suppressing NET formation. HQC directly binds MAPK14, inhibiting p38 MAPK phosphorylation and attenuating pathogenic neutrophil–FLS crosstalk (Li et al. 2025a, 2025b). Moreover, Simiao Yong'an Decoction (SMYAD) has also demonstrated potent anti‐arthritic effects by regulating neutrophil dynamics and directly suppressing neutrophil migration, ROS production, and NET formation in vitro (Jie et al. 2023).

Taken together, these findings suggest that regulation of NETosis represents another important mechanism by which TCM interrupts the amplification loop between innate immune activation, synovial inflammation, and joint injury in RA.

### 5.11 Cellular Senescence

In a comprehensive study integrating bioinformatics and machine learning approaches, Zheng et al. (2025) have identified MMP9, CXCL10, IL15, and FOXO3 as shared biomarkers linking RA, cellular senescence, and autophagy. Specifically, multi‐algorithm screening and experimental validation reveal that these genes are significantly dysregulated in RA and exhibit strong diagnostic efficacy. Notably, FOXO3 is downregulated and negatively correlated with other biomarkers. This study further demonstrates that taurine stabilizes FOXO3 through molecular docking and dynamics simulations; in vitro experiments confirm that taurine upregulates FOXO3 and ameliorates RA through the FOXO3‐Parkin pathway. This work provides novel insights into RA pathogenesis by regulating senescence‐associated genes, highlighting FOXO3 as a promising therapeutic target related to senescence and taurine as a potential RA treatment strategy.

### 5.12 Gut Microbiota

TCM, with its multi‐component and multi‐target characteristics, exerts therapeutic effects on RA partially through modulating the gut microbiome. It can enrich beneficial bacteria, suppress pathobionts, restore microbial diversity, and reinforce intestinal barrier integrity (Wang, Fu, et al. 2024; Wang, Kong, and Li 2024; Wang, Liu, et al. 2024; Wang, Xu, et al. 2024). Consequently, this modulation mitigates systemic inflammation and immune dysregulation by regulating the production of microbial metabolites (e.g., SCFAs) and influencing host immune responses (Peng et al. 2022). Emerging evidence from inflammatory disease research further supports that intestinal dysbiosis can aggravate disease progression through alterations in host–microbial fatty acid metabolism, thereby amplifying immune and metabolic disturbances (Zhao, Tang, et al. 2023; Zhao, Yu, et al. 2023). Thus, targeting the intestinal flora may be an effective strategy for RA treatment.

#### 5.12.1 TCM Monomer

Bai et al. (2025) have reported that Coix seed oil (CSO) demonstrate significant potential in alleviating RA‐associated sarcopenia by modulating the gut microbiota‐muscle axis. CSO administration can restore gut microbial homeostasis, enriching beneficial genera (e.g., Lactobacillus and Limosilactobacillus), while suppressing pro‐inflammatory pathways. These changes correlate with reduced systemic inflammation, improved muscle morphology, and inhibition of the JAK–STAT signaling; these findings underscore the therapeutic role of herbal interventions in RA muscle atrophy through microbiome regulation. Consistently, another study by Liu, Li, et al. (2025), Liu, Wang, et al. (2025), and Liu, Zhang, et al. (2025) has also proved that Fuzi ameliorates cold‐type RA by modulating gut microbiota (e.g., Lachnospiraceae and Ruminococcaceae) and microbial bile acid metabolism. Its anti‐inflammatory effects are mediated through metabolites [e.g., taurohyodeoxycholic acid (THDCA)], which activates the TGR5‐cAMP‐protein kinase A (PKA) pathway and suppresses NLRP3 inflammasome activation, revealing a microbiota‐metabolite‐immunity axis in RA treatment. Similarly, Cannabidiol (CBD), a herbal‐derived component, has also been shown to alleviate RA by modulating gut microbiota (e.g., *Allobaculum* and *Prevotella*), leading to increased production of butyric acid and L‐carnitine, which attenuate inflammation in immune and synovial cells (Geng et al. 2025).

#### 5.12.2 TCM Compound

It has been demonstrated that WTD alleviates adjuvant‐induced arthritis (AIA) in rats by modulating gut microbiota (e.g., *Bacteroides*, *Prevotella*, *Akkermansia*) and microbial metabolites, including SCFAs, bile acids, and tryptophan derivatives (Cheng et al. 2022). These changes correlate with reduced inflammation and improved gut barrier function, revealing a microbiota‐metabolite mechanism underlying its therapeutic effects. Similarly, Danggui Sini decoction (DSD) has also been shown to ameliorate CIA rats through multi‐pathway modulation, as revealed by ^1^H‐NMR metabolomics (Cheng et al. 2017). Specifically, DSD rectifies dysregulations in key metabolites (e.g., taurine, betaine, pyruvate) and associated pathways (including taurine metabolism, gut microbiota metabolism, and energy cycles), demonstrating its synergistic therapeutic mechanism. Another study by Liu et al. has revealed that Zhubi Decoction (ZBD) exerts significant anti‐arthritic effects in CIA rats through a multi‐target mechanism. It inhibits the PI3K/AKT pathway, rectifies 170 serum metabolic disturbances (particularly amino acids and lipids), and alleviates gut microbiota dysbiosis (Liu, Li, et al. 2025; Liu, Wang, et al. 2025; Liu, Zhang, et al. 2025). Additionally, Zushima Tablet (ZT) has been shown to alleviate RA in CIA rats by rectifying metabolic disturbances, notably in the TCA cycle, fatty acid, and purine metabolism (Shan et al. 2018). It regulates key serum and fecal metabolites, as well as SCFAs, while modulating gut microbiota (e.g., *Firmicutes*, *Bacteroidetes*), demonstrating a multi‐faceted mechanism of action. Consistent with this multi‐targeted approach, Duzheng Tablet (DZGP) ameliorates RA in mice by modulating synovial transcriptomics and gut microbiota (Zhao et al. 2024). Integrated multi‐omics analysis reveals that DZGP suppresses B cell‐mediated immunity and pro‐inflammatory cytokines (such as TNF‐α). It also concurrently restores gut ecological balance by normalizing the *Firmicutes*/*Bacteroidota* ratio and enriching beneficial phyla such as *Verrucomicrobiota* and *Cyanobacteria*. Furthermore, New Bitongling (NBTL) demonstrates efficacy in CIA rats by modulating gut microbiota (e.g., *f_Mycoplasmataceae*, *g_Prevotellaceae*) and suppressing angiogenesis (Guan et al. 2025). It inhibits the VEGF/VEGFR1/VEGFR2/HIF‐1α signaling axis and upregulates miR‐20a‐5p and miR‐223‐3p, providing a multi‐faceted mechanism for alleviating RA. These studies have further revealed the potential mechanisms of TCM in treating diseases, especially its unique advantages in regulating intestinal flora and improving RA.

## 6 Clinical Study on the Treatment of RA With TCM

Clinical studies on TCM for RA treatment, including rigorously designed randomized controlled trials (RCTs) and cohort studies, have generated substantial evidence supporting its efficacy and safety (summarized in Table 3). Additionally, data mining techniques applied to real‐world clinical datasets have identified potential herb combinations and underlying therapeutic mechanisms in RA; these findings facilitate further hypothesis‐driven research and methodological advancements. Clinical studies provide robust evidence for TCM's efficacy, bridge traditional practice with modern scientific validation, optimize clinical decision‐making, and illuminate novel drug discovery pathways for improved RA management.

**TABLE 3:** Clinical study on TCM for RA.

Research methods	TCM	Herbal composition	Sample size	Research methods	Dosage regimen	Primary endpoint	Outcome
RCT	XFC	Astragali Radix, Coicis Semen, Tripterygii Wilfordii Radix, and Scolopendra	Totally 304 RA patients were assigned to two groups: treatment group (152 cases) was administered XFC plus the placebo of LEF and control group (152 cases) given LEF plus the placebo of XFC for 12 weeks.	A multi‐center parallel‐group double‐blind RCT	Treatment group: XFC and placebo (for LEF). XFC: Three each time, 3 times a day immediately after meals, oral, for 3 months; placebo (for LEF): 10 mg each time, 1 time a day immediately after meal, oral, for 3 months. Control group: LEF and placebo (for XFC). LEF: 10 mg each time, one time a day immediately after meal, oral, for 3 months, placebo (for XFC): three each time, 3 times a day immediately after meals, oral, for 3 months.	ACR20, ACR50, ACR70, CRP, ESR, RF, anti‐CCP, IgG, IgA, IgM, HAQ, RAQOL, SAS, SDS	After 12‐week treatment, patients in two groups all showed some trend of effectiveness when compared in terms of ACR recommended 20%, 50%, 70% improvement criteria, but it was insignificant. The validity in ameliorate modified DAS28 and laboratory indexes as ESR, CRP, RF were also found no difference. The score of HAQ, SAS, SDS and RAQOL both lower than the first week and the changes showed no difference. However, the score of SDS dropped more in XFC group than in the other. A total of 147 adverse reaction cases were reported, which shows no difference between the two groups. The most common adverse reactions were hepatic impairment, anemia, leukocytopenia, epigastric discomfort and phalacrosis.
	XFC	Astragali Radix, Coicis Semen, Tripterygii Wilfordii Radix, and Scolopendra	The XFC + LEF placebo group (152 cases) and the LEF + XFC placebo group (152 cases), with a treatment duration of 12 weeks	A multi‐center parallel‐group double‐blind RCT	Treatment group: XFC and placebo (for LEF). XFC: Three each time, 3 times a day immediately after meals, oral, for 3 months; placebo (for LEF): 10 mg each time, 1 time a day immediately after meal, oral, for 3 months. Control group: LEF and placebo (for XFC). LEF: 10 mg each time, one time a day immediately after meal, oral, for 3 months, placebo (for XFC): three each time, 3 times a day immediately after meals, oral, for 3 months.	ACR20, ACR50, ACR70, DAS28, RAQOL, HAQ, SAS, SDS, APTT, TT, FBG, DD, PLT, PCT, PDW, MPV, ESR, CRP, RF, IgA, IgG, IgM, and CCP	The SDS score in the XFC group decreased more than in LEF group. XFC outperformed LEF in improving FBG, PLT, PCT, PDW, and MPV. A total of 147 adverse event cases were reported, with no significant difference between the two groups. The most common adverse reactions included liver damage, anemia, leukopenia, upper abdominal discomfort, and hepatic necrosis.
	QRHXD	Atractylodes chinensis, Phellodendron amurense, Smilax glabra Roxb, Lonicera japonica Thunb, Astragalus membranaceus, Paeonia lactiflora Pall, Dioscorea futschauensis Uline ex R. Kunth, Salvia miltiorrhiza Bge, Curcuma phaeocaulis Val, Sinomenium acutum, Polistes olivaceous, and Scolopendra subspinipes mutilans L. Koch	A total of 468 cases were randomly divided into an TCM group (TCM plus MTX and HCQ placebos) (156 cases), Western medicine (WM) group (156 cases) and integrative medicine (IM) group (156 cases) at a ratio of 1:1	A multi‐center parallel‐group double‐blind RCT	TCM group (QRHXD plus MTX and HCQ placebos); WM group (12.5 mg MTX once weekly plus 200 mg HCQ twice daily combined with TCM placebo); and IM group (TCM combined with 12.5 mg MTX once weekly plus 200 mg HCQ twice daily)	DAS28, ACR 20/50/70, TRAE, HAQ, ESR, CRP	QRHXD is effective for the treatment of patients with active RA, with a better safety profile than MTX and HCQ. While the efficacy of QRHXD alone was lower than that of the two csDMARDs, combining QRHXD with MTX and HCQ yielded the greatest improvement in disease activity. The efficacy of QRHXD alone was similar to that of MTX + HCQ in achieving ACR‐20/50/70.
	QRHXD	Atractylodes chinensis, Phellodendron amurense, Smilax glabra Roxb, Lonicera japonica Thunb, Astragalus membranaceus, Paeonia lactiflora Pall, Dioscorea futschauensis Uline ex R. Kunth, Salvia miltiorrhiza Bge, Curcuma phaeocaulis Val, Sinomenium acutum, Polistes olivaceous, and Scolopendra subspinipes mutilans L. Koch	A total of 204 cases were randomly divided into an QRHX granules plus MTX (102 cases) or a placebo plus MTX (102 cases) for 24 weeks. During the trial, 11 participants in the QRHX group and 15 in the placebo group dropped out; 91 (89.22%) participants in the QRHX group and 87 (85.29%) participants in the placebo group completed the study	A multicenter, randomized, double‐blind, placebo‐controlled trial	Participants in the QRHX group received QRHX granules 10 g twice daily plus MTX 10 mg once a week. Participants in the placebo group received QRHX placebo granules 10 g twice daily plus MTX 10 mg once a week.	BME score, bone erosion scores, DAS28	QRHX granules, combined with MTX, significantly reduced MRI‐detected BME and slowed bone erosion in active RA
	JBP	Drynariae Rhizoma, Rehmanniae Radix Praeparata, Angelicae Sinensis Radix, Cynanchi Paniculati Radix et Rhizoma, Eupolyphaga Steleophaga, Bombyx Batryticatus, Scolopendra, Scorpio, Vespae Nidus, Pheretima, Zaocys, Corydalis Rhizoma, Pyrolae Herba, Epimedii Folium, Aristolochiae Mollissimae Herba, Erodii Herba Geranii Herba, Spatholobi Caulis, Humuli Scandentis Herba, Rehmanniae Radix, and Polygoni Cuspidati Rhizoma et Radix	A total of 115 cases were randomly divided into JBP group (57 cases) and placebo group (58 cases) in a 1:1 ratio	A multicentre, randomized, double‐blind, placebo‐controlled trial	JBP (4 g, twice a day, orally) combined with MTX (10 mg per week) or placebo (4 g, twice a day, orally) combined with MTX for 12 weeks	DAS28‐ESR, DAS28‐CRP, ACR20/50, HAQ‐DI, CDAI, VAS, SF‐36, MOS, ESR, CRP	Compared to the placebo group, the JBP group demonstrated significantly greater improvements in the primary endpoints (CDAI and HAQ‐DI scores) and key secondary outcomes (VAS, ESR, CRP levels, ACR20/50 response rates, and DAS28‐ESR/CRP) at both the 12‐week and 48‐week marks. No significant differences in the incidence of adverse events were observed between the two groups during the treatment period.
Cohort study	Xin'an Jianpi Tongbi prescription	Astragalus membranaceus, Semen coicis, Tripterygium wilfordii, Scolopendra spp., Scutellaria baicalensis, Gardenia jasminoides, Prunus persica, Clematis chinensis, Poria cocos, Epimedium brevicornu, Cinnamomum cassia, Curcumae Longae, and other drugs	A total of 11,074 patients with RA were included in the study	A retrospective cohort study	Received Xin'an Jianpi Tongbi prescription for less than 1 month	ESR, CRP, anti‐CCP, RF, IgA, IgG, IgM, C3, C4, readmission, Sjogren's syndrome, surgical treatment, and all‐cause death	Long‐term exposure to Xin'an Jianpi Tongbi prescription in patients with RA, decreased the risk of RA‐related complications, including readmission, Sjogren's syndrome, surgical treatment, and all‐cause death.
	TCM	Poria, Glycyrrhizae Radix et Rhizoma, Salvia miltiorrhiza Bunge, Carthami flos, Citri Reticulatae Pericarpium, Coicis Semen, Dioscoreae Rhizoma, Persicae Semen, Taraxaci Herba, Clematidis Radix et Rhizoma, Siegesbeckiae Herba, Hedyotis diffusa, Alismatis rhizoma, Spatholobi Caulis, Pinelliae Rhizoma Praeparatum cum Zingibere et Alumine	A total of 1665 patients with RA were included in the study	A retrospective study	Received TCM for less than 3 month	ALT, AST, ESR, CRP, PLT, IgM, IgG, IgA, C3, C4, and RF	TCM is associated with lower risk of recurrent exacerbation and may increase survival time in RA patients
	TCM	Safflower, Salvia, Spatholobus suberectus Dunn, Poria, Coix Seed, Chinese yam, Radix Clematis, Herba Siegesbeckiae, Herba Lycopodii, Dandelion, Caulis Lonicerae, Hedyotis diffusa	A total of 1265 patients with RA were included in the study	A retrospective cohort study	Received TCM for less than 1 month	LT, D‐D, FIB, TT, APTT, RF, CCP, IgA, IgG, IgM, C3, C4, ERS, hs‐CRP, ASO, NEU, LYM, NLR, ALT, AST, CREA, BUN, UA	TCM as a protective factor capable of reducing the risk of readmission and alleviating laboratory indicators in RA‐HC patients. Moreover, the longer the duration of TCM intervention, the lower the risk of readmission and the more significant the efficacy.
	TCM	Carthami Flos, Persicae Semen, Liquidambaris Fructus, Spatholobi Caulis, Lycopodii Herba, Clematidis Radix et Rhizoma, Chuanxiong Rhizoma, Lonicerae Japonicae Caulis, Taraxaci Herba, Salviae Miltiorrhizae Radix et Rhizoma, Violae Herba, Siegesbeckiae Herba, Hedyotis Diffusae Herba, Coicis Semen, Magnoliae Officinalis Cortex, Citri Reticulatae Pericarpium, Poria, Glycyrrhizae Radix et Rhizoma, Pinelliae Rhizoma, and Dioscoreae Rhizoma	A total of 2437 patients with RA were included in the study	A retrospective cohort study	Received TCM for less than 1 month	ALB, TP, ESR, CRP, RF, CCP, IgA, IgG, IgM, C3, C4	On the basis of western medicine treatment, the application of TCM could reduce the readmission rate of RA patients with hypoproteinemia, and longer use of TCM indicated lower readmission rate
	TCM	Poria, Coicis Semen, Hordei Fructus Germinatus, Pinelliae Rhizoma, Oryzae Fructus Germinatus, Citri Reticulatae Pericarpium, Dioscoreae Rhizoma, Astragali Radix, Angelicae Sinensis Radix, Spatholobi Caulis, Salviae Miltiorrhizae Radix et Rhizoma, Persicae Semen, Carthami Flos, Chuanxiong Rhizoma, Taraxaci Herba, Hedyotis Diffusae Herba, Angelicae Pubescentis Radix, Lycopodii Herba, and Clematidis Radix et Rhizoma	A total of 883 patients with RA were included in the study	A retrospective cohort study	Received TCM for less than 3 month	ESR, hs‐CRP, RF, CCP, IgA, IgG, IgM, C3, C4,interstitial lung disease, Sjögren's syndrome, and anemia	Long‐term TCM treatment on reducing the occurrence time and probability of extra‐articular lesions in RA patients, including interstitial lung disease, Sjögren's syndrome, and anemia
	XFC	Astragali Radix, Coicis Semen, Tripterygii Wilfordii Radix, and Scolopendra	A total of 9987 patients with RA were included in the study	A Cohort Study	Received TCM for less than 12 month	ESR, CRP, RF, IgA, IgG, IgM, C3, C4, PLT	XFC as a remarkable protective factor that not only mitigates the risk of readmission and prolongs survival among RA patients but also markedly ameliorates their clinical immune‐inflammatory biomarkers
	HQC	Scutellariae Radix, Coicis Semen, Persicae Semen, Gardeniae Fructus, and Clematidis Radix et Rhizoma	A total of 3423 patients with RA were included in the study	A real‐world retrospective cohort study	Received TCM for less than 1 month	CRP, RF, CCP, IgA, IgG, IgM, C3, C4, ALT, AST. CREA, BUN, UA, SF‐36, VAS, SAS, SDS, CMSS‐RADHP	HQC treatment, as a protective factor, is associated with the reduction of the risk of hospital readmission in patients with RA. Furthermore, HQC can significantly improve the immunoinflammatory indicators of patients without the risk of hepatorenal toxicity. Importantly, HQC treatment can improve SPP and improve QOL and satisfaction of patients with RA
Data mining	TCM	Poria, Pericarpium Citri Reticulatae, Semen Coicis, Fructus Hordei Germinatus, Radix Salviae Miltiorrhizae, Flos Carthami, Semen Persicae, Caulis Spatholobi, and Rhizoma Chuanxiong, Radix et Rhizoma Clematidis Chinensis, Sigesbeckia orientalis, Rhizoma Alismatis, Semen Plantaginis, and Radix Angelicae Biserratae, Herba Taraxaci Mongolici, Herba Hedyotdis, Radix Scutellariae Baicalensis, Cortex Phellodendri Amurensis, and Rhizoma Anemarrhenae	A total of 9480 patients with RA were included in the study	Retrospective clinical data mining	Received TCM for less than 10 days	CRP, ESR, CCP, RF, IgA, IgM, IgG, C3, C4	There are complementary relationships among the constituent herbs in Chinese herbal medicine used to treat RA, which produce different therapeutic effects in RA treatment
	Different dosage forms of Chinese herbal medicines	Poria Cocos, Flos Carthami, Salvia Miltiorrhiza, Pericarpium Citri Reticulatae, Coix Seed, Glycyrrhiza uralensis Fisch, Herba Taraxaci, Semen Pruni Persicae, Rhizoma Dioscoreae, Radix Clematidis, Herba Siegesbeckiae, Caulis Spatholobi, Fructus Hordei Germinatus, and Rhizoma Alismatis	A total of 6829 patients with RA were included in the study	A multicenter, retrospective clinical data mining study	Received TCM for less than 10 days	RF, CCP, ESR, hs‐CRP, IgA, IgG, C3, C4, ALT, AST, BUN and CREA	Granules and decoction pieces of Chinese herbal medicines have similar efficacy in improving immune‐inflammatory indices in RA patients and could be used as treatment options for RA
	TCM	Jinyinhua, Yiyiren, Wugong, Qingfengteng, Chaobaishao, Tusizi, Baizhu, Fuzi, Guizhi, Qianghuo, Tufuling, Fangji, Sangjisheng, Shuizhi, Huainiuxi, and Duhuo	A total of 311 patients with RA were included in the study	Retrospective clinical data mining	Received TCM for less than 10 days	Symptoms of “Chronic pain”	Four potential analgesic herbs (“Wugong,” “Qingfengteng,” “Jinyinhua,” and “Yiyiren”) with their combinations may help to alleviate RA pain with moderate activity.
	Tripterygium Glycosides Tablets and TCM	Tripterygium Glycosides Tablets	A total of 1220 patients with RA were included in the study	Retrospective clinical data mining	Received TCM for less than 10 days	ESR, RF, CCP, hs‐CRP, IgA, IgM, IgG, ALT, AST, ALP, GGT, BUN, CREA, UA, b‐MG, MA, TRU, IgU	Combination therapy with Tripterygium Glycosides Tablets and TCM demonstrated superiority over Tripterygium Glycosides Tablets alone in improving immunoinflammatory parameters and reducing hepatorenal toxicity in RA patients. Furthermore, a long‐term association was observed between the improvement in these indices and the treatment regimen.

### 6.1 RCT

RCTs are the gold standard in clinical research, which minimize bias and provide the highest level of evidence for establishing causal treatment effects. Through RCTs, researchers can systematically evaluate treatment efficacy and safety, provide high‐quality evidence to support clinical decisions, and drive advancements in medical science.

The safety of TCM for RA treatment has also been validated in RCTs. For example, in a multi‐center, double‐blind RCT of 304 RA patients, XFC demonstrates comparable efficacy and safety to leflunomide (LEF) over 12 weeks (Liu et al. 2015). While no significant differences are observed in primary endpoints (ACR response, DAS28, ESR, and CRP), XFC shows superior amelioration of depressive symptoms, as measured by the Self‐Rating Depression Scale (SDS). Furthermore, a post hoc analysis of this RCT reveals that coagulation and platelet parameters are significantly correlated with RA disease activity and impaired quality of life (Wang, Liu, et al. 2025; Wang, Ni, et al. 2025; Wang, Pan, et al. 2025; Wang, Wen, et al. 2025; Wang, Zhang, et al. 2025). Notably, XFC demonstrates superior efficacy over LEF in improving key hemostatic parameters (including fibrinogen, platelet count, and platelet indices), suggesting a potential novel mechanism for its clinical benefits. In a multi‐center RCT of 468 active RA patients (NCT02551575), QRHX granules demonstrate significant efficacy in reducing DAS‐28, outperforming csDMARDs alone (Gong et al. 2021). Although ACR response rates are comparable across groups, the TCM regimen exhibits a significantly superior safety profile, highlighting its potential as a valuable and safer complementary option to conventional therapy. In a subsequent multicenter RCT (*n* = 204), QRHX granules combined with methotrexate (MTX) notably reduce MRI‐assessed bone marrow edema scores and slow bone erosion progression compared to placebo at 24 weeks, with a favorable safety profile (Gong et al. 2025). This provides high‐level evidence supporting its adjunctive use for protecting bone health in active RA patients. In another multi‐center RCT, Juan Bi Pill (JBP) combined with MTX effectively reduces disease activity (DAS28‐ESR/CRP), improves ACR20/50 response rates, and enhances physical function in active RA patients over 12 and 48 weeks, with a safety profile comparable to placebo (NCT02885597) (Jia et al. 2025).

RCTs of TCM in treating RA have not only validated its therapeutic efficacy and safety but also provided scientific evidence for its combination with modern medications. These findings enhance TCM's recognition within the global medical community and offer RA patients more treatment options and improved quality of life. As more high‐quality RCTs are conducted, TCM's role in RA treatment will become increasingly recognized and widely adopted.

### 6.2 Cohort Study

Cohort studies represent a cornerstone of clinical research, providing robust longitudinal data essential for elucidating causal inferences between exposures and outcomes. Their observational yet prospective nature allows for direct assessment of disease incidence and natural history, offering high‐quality evidence that bridges experimental findings and real‐world clinical practice.

According to a retrospective cohort study (*n* = 11,074), TCM compound preparations (TCMCPs) significantly reduce the risk of RA‐related complications, including readmission, Sjögren's syndrome, surgery, and all‐cause mortality. Propensity score‐matched analysis reveals a dose‐dependent protective effect, with higher TCMCP exposure associated with a lower composite endpoint risk (HR = 0.75, 95% CI: 0.71–0.80) (Fang, Liu, and Wan 2023; Fang, Liu, Xin, et al. 2023). In another retrospective cohort study of 1383 RA patients, propensity score‐matched analysis reveals that TCM is remarkably associated with a reduced risk of recurrent exacerbation (HR = 0.50, 95% CI: 0.65–0.92, *p* < 0.01) (Wang, Liu, Fang, Li, et al. 2023; Wang, Liu, Fang, Wen, et al. 2023). K‐M analysis further demonstrates a superior survival rate for TCM users, providing evidence to support the recommendation of TCM treatment for RA patients. Furthermore, retrospective cohort investigations have demonstrated that TCM use is associated with a significant reduction in the incidence of extrar‐articular manifestations in RA patients. This provides compelling evidence for its protective role against systemic complications. For example, Li, Liu, et al. (2024) and Li, Wan, et al. (2024) have conducted a retrospective cohort study and found that TCM intervention for ≥ 1 month notably reduces readmission risk, surgical rates, and all‐cause mortality in RA patients with hypercoagulability, while also improving key laboratory indicators [e.g., RF, high‐sensitivity CRP (hs‐CRP), and NLR]. Another similar study has shown that TCM intervention for ≥ 1 month is associated with a significantly reduced readmission risk in RA patients with hypoproteinemia, suggesting that TCM is a protective factor and confirming that older age is a risk factor (Zhou et al. 2023). Further supporting this, Wen et al. have revealed that long‐term TCM intervention (≥ 3 months) remarkably delays the onset of extra‐articular lesions (e.g., interstitial lung disease and anemia) in RA patients. Multivariate analysis identifies TCM as a protective factor, whereas elevated IgG and C4 levels are risk factors for specific complications. These findings indicate that TCM can be applied flexibly throughout the treatment process for RA patients (Wen et al. 2023).

Furthermore, two additional cohort studies have specifically investigated the association between TCM compounds and the risk of readmission in RA patients. One of the studies indicates that XFC treatment is significantly associated with a reduced risk of readmission in RA patients (Wang, Fu, et al. 2024; Wang, Kong, and Li 2024; Wang, Liu, et al. 2024; Wang, Xu, et al. 2024). Using propensity score matching and Cox regression analysis, the study involving over 6000 patients identifies XFC as a protective factor, and longer treatment durations (> 12 months) yield more substantial benefits. Moreover, this study further links XFC usage to the improvements in key inflammatory and immunological markers (CRP, ESR, C3, and C4), underscoring the potential of XFC as a long‐term therapeutic strategy to improve patient outcomes. Another real‐world cohort study has demonstrated that HQC treatment noticeably reduces readmission risk and improves inflammatory markers (ESR, CRP, C3, and RF) and patient‐reported outcomes in RA patients; multivariate analysis further identifies HQC as a protective factor, with no associated hepatorenal toxicity (Hu et al. 2023).

Cohort studies hold an irreplaceable position in clinical research, particularly in the study of TCM treatment for RA. Therefore, they are not only important tools in clinical research but also vital pathways for promoting the modernization and internationalization of TCM.

### 6.3 Data Mining

The application of data mining in TCM treatment for RA can not only reveal potential mechanisms and drug combinations but also provide scientific evidence and decision‐making support for clinical practice. Furthermore, data mining applications in TCM treatment are particularly evident in predicting and optimizing drug efficacy. In RA management, researchers have developed multiple predictive models using data mining techniques to forecast treatment outcomes under different therapeutic approaches. These data‐driven predictive models not only enhance treatment precision but also pave the way for personalized medicine.

Fang et al. (2020) have employed a random walk model to analyze clinical data from 9408 RA patients, and they demonstrate that specific combinations of TCM components synergistically improve multiple clinical indicators, including ESR, CRP, C3, C4, and IgA; these effects are more effective than individual drugs alone. In the treatment of RA, data mining technology has provided new perspectives for studying prescription patterns of different TCM formulations. A retrospective data mining study of 6829 RA patients has revealed that both granule and decoction forms of core Chinese herbal prescriptions significantly improve immune‐inflammatory indices; these prescriptions are primarily used for spleen‐strengthening and blood‐activating therapies, with comparable efficacy and safety profiles (Huang et al. 2022). Lai et al. (2022) have employed the Apriori algorithm for data mining of 311 RA prescriptions and identified four potential analgesic herbs (*Lonicerae Japonicae Flos*, *Scolopendra*, *Coicis Semen*, and *Sinomenii Caulis*). Association rule analysis reveals 17 key rules, providing novel herbal combinations for effective clinical pain management in RA. Additionally, a clinical data mining study of 1220 RA patients has demonstrated that combining Tripterygium Glycosides with TCM could efficiently improve key immune‐inflammatory parameters (IgA, IgG, RF, CCP‐AB, hs‐CRP, and ESR) in RA patients compared to monotherapy, although a greater reduction in IgM is observed in the controls. The combination therapy also shows superior renoprotective effects and a more favorable profile in random walk model analysis (Dong et al. 2019).

In RA treatment, the use of data mining technology is particularly crucial. It helps identify effective drug combinations and offers new perspectives for modernizing TCM by uncovering multi‐target and multi‐pathway mechanisms of action.

### 6.4 Safety Considerations of TCM in RA Treatment

TCM has shown considerable therapeutic potential in RA; however, its safety profile requires balanced and critical consideration, particularly for long‐term use and combination with conventional DMARDs. Although many herbal formulas and monomeric compounds are generally well tolerated in clinical practice, certain TCM components (especially preparations containing *Tripterygium wilfordii* ) have been associated with well‐documented toxicities, including hepatotoxicity, nephrotoxicity, reproductive toxicity, gastrointestinal intolerance, and hematologic adverse effects (Ru et al. 2019). Additionally, some patients—such as pregnant women, individuals with impaired liver or kidney function, or those receiving multiple immunosuppressive agents—may require particular caution or even avoidance of specific TCM interventions (Feng et al. 2019). Moreover, potential herb–drug interactions should also be considered, as concurrent use with MTX, LEF, glucocorticoids, or biologic agents may alter drug metabolism, enhance toxicity, or complicate adverse event attribution (Sharma et al. 2022). Therefore, the safe clinical application of TCM in RA depends on multiple factors: efficacy evaluation, rigorous quality control, proper processing of herbal materials, standardized formulations, dose optimization, and careful monitoring of adverse reactions. Strengthening phytochemical standardization and pharmacovigilance will be essential to improve the safety, reproducibility, and global acceptability of TCM‐based therapies.

## 7 Conclusions and Perspectives

The therapeutic potential of TCM in RA is deeply grounded in its long‐standing traditional use for “Bi Zheng.” The multi‐target effects of TCM monomers and formulas provide modern validation of the empirical wisdom embedded in TCM practice. This integrative review systematically consolidates recent advances in understanding the therapeutic mechanisms and potential of TCM for RA management. Clinical, experimental, and bioinformatic evidence reveals that TCM—including monomeric compounds, herbal formulations, and patented prescriptions—exerts multi‐target, multi‐pathway effects that align with the complex etiopathology of RA (summarized in Graphical Abstract).

Importantly, these mechanisms should not be interpreted as independent or linear processes. Rather, RA is characterized by a highly interconnected pathogenic network. In this network, inflammation, oxidative stress, programmed cell death, angiogenesis, gut microbiota dysbiosis, and cellular senescence dynamically interact and mutually reinforce disease progression. This systems‐level perspective provides a conceptual framework for understanding the therapeutic advantages of TCM.

The following conclusions and future perspectives are drawn from the comprehensive analysis presented above.

### 7.1 Mechanistic Depth and Multi‐Target Action

TCM demonstrates a capacity to simultaneously modulate multiple pathological processes in RA, encompassing inflammation, apoptosis, oxidative stress, ferroptosis, pyroptosis, angiogenesis, hypercoagulability, NET formation, cellular senescence, and gut microbiota dysbiosis. Notably, the involvement of ncRNAs (e.g., lncRNAs, circRNAs, miRNAs) and epigenetic modifications (e.g., m6A methylation) in mediating these effects underscores the sophistication of TCM's action mechanism. These findings affirm that TCM acts on multiple targets, matching the complexity and heterogeneity of RA.

More importantly, increasing evidence suggests that these mechanisms are interconnected rather than isolated. For example, oxidative stress can act as an upstream driver that promotes lipid peroxidation and ferroptosis, while also accelerating cellular senescence. In turn, senescent cells amplify inflammatory responses through the SASP, thereby forming a feed‐forward loop that exacerbates RA progression. Similarly, inflammasome activation and pyroptosis further reinforce inflammatory signaling and tissue injury. Therefore, TCM‐mediated regulation of these pathways may simultaneously disrupt multiple pathological nodes within this interconnected network.

### 7.2 Clinical Translation and Evidence‐Based Validation

Rigorous clinical studies, including RCTs and large‐scale cohort analyses, have substantiated the efficacy and safety of TCM interventions. Some TCM Formulations (e.g., XFC, HQC, and QRHXD) have shown comparable or superior outcomes relative to conventional DMARDs, particularly in improving inflammatory markers, coagulation parameters, quality of life, and long‐term prognostic indicators. Real‐world data mining further supports the optimization of herbal combinations and the personalization of treatment strategies.

### 7.3 Synergy With Conventional Therapy

TCM exhibits potential as a complementary or alternative therapy, particularly for patients with inadequate responses to or intolerable side effects from biologic agents. TCM mitigates adverse effects, reduces relapse rates, and delays extra‐articular complications, highlighting its role in integrated treatment paradigms. Moreover, its favorable safety profile also positions TCM as a viable option for long‐term management.

### 7.4 Limitations and Research Gaps

Despite these encouraging findings, several challenges remain. Many mechanistic studies rely on preclinical models, necessitating further validation in human systems. Standardization of herbal extracts, quality control of formulations, and reproducibility of findings across different populations are critical for clinical applicability. Additionally, the active components of multi‐herb formulations and their pharmacokinetic interactions require further investigation.

In particular, batch‐to‐batch consistency remains a major challenge for the modernization and clinical translation of TCM formulations. Future studies should incorporate metabolomics‐based quality control strategies to characterize herbal chemical profiles, identify representative biomarkers, and ensure batch‐to‐batch consistency. Such approaches may help improve standardization, reproducibility, and reliability in both experimental and clinical settings.

### 7.5 Future Directions

#### 7.5.1 Ethnopharmacology‐Driven Drug Discovery

Future research should prioritize detailed documentation of traditional use. Plants with a strong history of use for specific RA syndromes should be prioritized. This approach offers a more efficient way to identify novel lead compounds and understand synergistic effects in multi‐herb formulations.

#### 7.5.2 Mechanistic Elucidation

Advanced omics technologies (e.g., single‐cell sequencing, spatial transcriptomics, and metabolomics) should be employed to decipher the precise molecular networks through which TCM modulates RA pathology.

#### 7.5.3 Clinical Trial Design

Future RCTs should include biomarker‐driven stratification, longer follow‐ups, and direct comparisons with advanced biologics to clarify TCM's role in RA treatment guidelines.

In addition, the integration of artificial intelligence (AI), machine learning, and systems pharmacology may provide a practical framework for advancing TCM‐based precision medicine in RA. Rather than remaining at the level of general conceptual discussion, these approaches could be applied to representative formulas discussed in this review, such as Xinfeng Capsule (XFC). For example, network pharmacology could be used to construct compound‐target‐pathway interaction networks for XFC and to identify key regulatory nodes involved in inflammation, oxidative stress, apoptosis, pyroptosis, ferroptosis, angiogenesis, and gut microbiota‐related pathways. On this basis, machine learning models could be introduced to predict potentially synergistic combinations among multiple herbal components, distinguish core therapeutic modules from supportive components, and prioritize the most informative candidate targets for subsequent experimental validation. In parallel, integration with multi‐omics data, including transcriptomics, metabolomics, and single‐cell sequencing, may help validate these predicted interactions, improve mechanistic interpretability, and identify patient subgroups that are more likely to benefit from specific TCM interventions. Furthermore, metabolomics‐based quality control strategies may be used to characterize the chemical fingerprints of formulations such as XFC, thereby improving batch‐to‐batch consistency and enhancing clinical reproducibility. Coupled with nanotechnology‐enabled delivery systems and biomarker‐guided validation, these interdisciplinary strategies may help move TCM from phenomenological observation toward mechanism‐guided and precision‐oriented medicine. At the same time, aligning TCM research with international standards, including CONSORT (Consolidated Standards of Reporting Trials)‐guided trial reporting and rigorous phytochemical standardization, will be essential for improving scientific credibility and promoting global acceptance.

In conclusion, this review summarizes the multi‐target mechanisms and clinical evidence of TCM for RA. More importantly, it establishes a robust link between the traditional use of medicinal plants and their contemporary pharmacological validation. The bioactive compounds and effective formulas discussed here are not isolated discoveries but scientific explanations for the efficacy observed in centuries of TCM practice against “Bi Zheng.” Overall, the therapeutic paradigm of TCM reflects a systems‐oriented strategy that is particularly well suited for complex diseases (such as RA), in which multiple pathogenic processes are tightly interconnected.

## Author Contributions

**Jianting Wen:** writing – original draft, resources. **Jian Liu:** conceptualization, project administration, funding acquisition, supervision. **Lei Wan:** methodology, writing – review and editing. **Yang Li:** formal analysis, investigation. **Fanfan Wang:** data curation, software.

## Funding

This work was supported by the National Natural Science Foundation of China (no. 82505489), Natural Science Foundation of Anhui Province (no. 2508085QH332), Clinical Research Project of Anhui University of Traditional Chinese Medicine in 2024 (2024YFYLCZX09), Anhui University of Chinese Medicine 2024 Annual University‐Level Exploratory Research Project (AHUCM2024TS099), and Anhui Provincial Research Project on Traditional Chinese Medicine Inheritance and Innovation (No. 2025CCCX005).

## Conflicts of Interest

The authors declare no conflicts of interest.

## Data Availability

Data sharing not applicable to this article as no datasets were generated or analyzed during the current study.
